# Transcriptional Patterns in Peritoneal Tissue of Encapsulating Peritoneal Sclerosis, a Complication of Chronic Peritoneal Dialysis

**DOI:** 10.1371/journal.pone.0056389

**Published:** 2013-02-13

**Authors:** Fabian R. Reimold, Niko Braun, Zsuzsanna K. Zsengellér, Isaac E. Stillman, S. Ananth Karumanchi, Hakan R. Toka, Joerg Latus, Peter Fritz, Dagmar Biegger, Stephan Segerer, M. Dominik Alscher, Manoj K. Bhasin, Seth L. Alper

**Affiliations:** 1 Renal Division, Beth Israel Deaconess Medical Center, Harvard Medical School, Boston, Massachusetts, United States of America; 2 Department of Medicine, Harvard Medical School, Boston, Massachusetts, United States of America; 3 Department of General Internal Medicine and Nephrology, Robert-Bosch Hospital, Stuttgart, Germany; 4 Institute of Digital Medicine, Stuttgart, Germany; 5 Department of Pathology, Beth Israel Deaconess Medical Center and Harvard Medical School, Boston, Massachusetts, United States of America; 6 Center for Vascular Biology Research, Beth Israel Deaconess Medical Center and Harvard Medical School, Boston, Massachusetts, United States of America; 7 Division of Nephrology and Department of Medicine, Brigham and Women's Hospital and Harvard Medical School, Boston, Massachusetts, United States of America; 8 Division of Pathology, Department of Diagnostic Medicine, Robert-Bosch Hospital, Stuttgart, Germany; 9 Margarete Fischer-Bosch Institute of Clinical Pharmacology, Stuttgart, Germany; 10 Division of Nephrology, University Hospital Zurich, Zurich, Switzerland; 11 Division of Interdisciplinary Medicine and Biotechnology and Department of Medicine, Beth Israel Deaconess Medical Center and Harvard Medical School, Boston, Massachusetts, United States of America; University of Massachusetts Medical, United States of America

## Abstract

Encapsulating peritoneal sclerosis (EPS) is a devastating complication of peritoneal dialysis (PD), characterized by marked inflammation and severe fibrosis of the peritoneum, and associated with high morbidity and mortality. EPS can occur years after termination of PD and, in severe cases, leads to intestinal obstruction and ileus requiring surgical intervention. Despite ongoing research, the pathogenesis of EPS remains unclear. We performed a global transcriptome analysis of peritoneal tissue specimens from EPS patients, PD patients without EPS, and uremic patients without history of PD or EPS (Uremic). Unsupervised and supervised bioinformatics analysis revealed distinct transcriptional patterns that discriminated these three clinical groups. The analysis identified a signature of 219 genes expressed differentially in EPS as compared to PD and Uremic groups. Canonical pathway analysis of differentially expressed genes showed enrichment in several pathways, including antigen presentation, dendritic cell maturation, B cell development, chemokine signaling and humoral and cellular immunity (P value<0.05). Further interactive network analysis depicted effects of EPS-associated genes on networks linked to inflammation, immunological response, and cell proliferation. Gene expression changes were confirmed by qRT-PCR for a subset of the differentially expressed genes. EPS patient tissues exhibited elevated expression of genes encoding sulfatase1, thrombospondin 1, fibronectin 1 and alpha smooth muscle actin, among many others, while in EPS and PD tissues mRNAs encoding leptin and retinol-binding protein 4 were markedly down-regulated, compared to Uremic group patients. Immunolocalization of Collagen 1 alpha 1 revealed that Col1a1 protein was predominantly expressed in the submesothelial compact zone of EPS patient peritoneal samples, whereas PD patient peritoneal samples exhibited homogenous Col1a1 staining throughout the tissue samples. The results are compatible with the hypothesis that encapsulating peritoneal sclerosis is a distinct pathological process from the simple peritoneal fibrosis that accompanies all PD treatment.

## Introduction

Renal replacement therapy is currently restricted to renal transplantation, hemodialysis (HD), and peritoneal dialysis (PD). Peritoneal dialysis constitutes <10% of current renal replacement therapy in the US and in much of Europe, but up to 80% in Mexico and Taiwan [1]. However, reimbursement changes expected to accompany health care reform in the US will promote renewed interest in and increased use of PD.

Encapsulating peritoneal sclerosis (EPS) is a rare but dangerous complication of peritoneal dialysis (PD). Mortality rates as high as 57% [2], [3] have been reduced at centers specializing in the medical and surgical treatment of EPS, even in late stage disease characterized by severe intestinal obstruction [3], [4], [5]. Although sporadic idiopathic EPS has been reported [6], [7], [8], prior duration of PD remains the most important risk factor identified to date [2], [9]. EPS epidemiology has been complicated by regional differences in PD use and in reported EPS incidence, likely exacerbated by non-uniform diagnostic criteria. EPS rates among 7000 patients from Australia and New Zealand were 0.3% after 3 years on PD, 0.8% after 5 years, and 3.9% after 8 years [9], but 8.1% of UK patients treated with PD for 5 years developed EPS [10]. In a Japanese cohort of PD patients with overall EPS incidence of 2.5%, EPS was diagnosed in 17–70% of patients with PD duration >15 years [11], [12], [13]. 72% of EPS is recognized only after discontinuation of PD due to ultrafiltration failure, switch to hemodialysis, or transplantation [9]. Among transplanted PD patients in the Dutch multicenter EPS study, EPS was the fourth most common cause of death after infection, cardiovascular disease, and malignancy [14].

The pathogenesis of EPS remains incompletely understood. Simple peritoneal fibrosis accompanies nearly all PD treatment, resulting in gradual impairment of ultrafiltration that can necessitate transition to HD [15]. Risk factors and signaling pathway abnormalities distinguishing the malignant fibrotic process of EPS from simple peritoneal fibrosis are poorly defined. The current two-hit model envisions as a first hit the long-term exposure to advanced glycation end products (AGEs) in peritoneal dialysate [16], [17], leading to increased expression of profibrotic factors such as transforming growth factor β (TGF-β) and of angiogenic mediators such as vascular endothelial growth factor (VEGF). In addition, declining numbers of pro-fibrinolytic mast cells promote enhanced fibrin deposition [18]. A contributing role has also been proposed for the turbulent fluid shear stress intrinsic to the process of PD [19], [20]. The second hit remains unknown, but may be a clinically obvious or occult inflammatory or ischemic stimulus. Peritoneal mesothelial cells are believed to undergo epithelial-to-mesenchymal transition (EMT), leading in EPS to complete mesothelial denudation that accompanies the severe fibrosis [21], [22]. However, the high rates of EPS among patients treated with PD over lengthy periods also suggest an alternate hypothesis. EPS may instead represent the natural evolution of PD-associated peritoneal fibrosis, influenced by patient-specific risk modifier gene profiles that determine the kinetics of progression from simple fibrosis to EPS.

The absence of blood tests specific for EPS requires diagnosis based on clinical presentation, radiologic and histologic findings [2]. The clinical presentation of EPS is characterized by varied and nonspecific symptoms, including bowel obstruction, loss of appetite, fever, nausea and vomiting, ascites, constipation, diarrhea and weight loss. The diagnosis of EPS is most commonly made by CT scan, but the radiological picture can be nonspecific [23]. Diagnosis also can be made by peritoneoscopy or laparotomy, classically revealing abdominal cocooning (bands or layers of fibrotic tissue surrounding and constricting bowel loops), sometimes accompanied by a fibrotic “sugar coating” appearance. Histologically, EPS peritoneal tissues often contain myofibroblast-like cells expressing smooth muscle actin-1 and podoplanin [22]. Braun et al. have recently proposed additional novel histological criteria for the diagnosis of EPS, including mesothelial denudation, fibrin deposits, and presence of fibroblast-like cells [24].

Non-surgical therapeutic options for EPS are few, and randomized controlled trials non-existent [12]. Glucocorticoids have been used, especially in settings of marked inflammation [12], [25], [26], [27]. Clinical responses to azathioprine or mycophenolate have been reported [28], but the increased post-transplant prevalence of EPS suggests possible deleterious, profibrotic actions of calcineurin inhibitors [29]. Anecdotal reports of beneficial treatment with tamoxifen have been attributed to inhibition of profibrotic TGF-β [30], [31]. Inhibitors of the renin angiotensin aldosterone system (RAAS), widely used in PD patients and including angiotensin converting enzyme inhibitors (ACEi) and angiotensin receptor blockers (ARB), have been proposed to deter development of EPS in PD patients [32]. Indeed, RAAS inhibition in rat models has reduced angiogenesis and peritoneal thickening [32], and retarded or reduced progression from simple fibrosis to a condition resembling EPS [33]. However, severe EPS cases characterized by enteral obstruction or ileus usually require urgent surgical enterolysis and debridement. Although the outcomes of acute surgical intervention are often favorable, EPS recurs in up to 23% of these post-surgical patients [12], [34].

Models of peritoneal fibrosis in rats and mice [35] have been generated by peritoneal insertion of foreign bodies [36], by peritoneal instillation of peritoneal dialysate containing glucose oxidation products for periods up to 3 weeks [37], by peritoneal instillation of inflammatory agents such as chlorhexidine gluconate [38], and by adenovirus-driven overexpression of TGF-β1 [39]. These models have offered opportunities for unbiased examinations of changes in global gene expression [36], [37], [38] that might shed light on the pathogenesis of peritoneal fibrosis. However the relationships between these short-term rodent models and the human conditions of PD-associated slowly progressive peritoneal fibrosis or the more serious and aggressive EPS remain unclear.

Therefore, we have performed a pilot study to compare transcriptomes of fresh-frozen peritoneal biopsy samples from EPS patients with those of PD patients without EPS, and with those of uremic patients (Uremic) prior to initiation of dialysis. We employed systems biology and interactive network analyses to identify pathways and interactive networks enriched with EPS-dysregulated genes, to establish the feasibility of using this approach to unravel pathophysiological mechanisms of EPS development in PD patients. This pilot study lays an empiric foundation for future investigations to understand biological mechanisms of EPS and to identify novel prognostic and therapeutic biomarkers.

## Methods

### Sample accrual

All samples were obtained from the peritoneal biopsy registry at Robert-Bosch Hospital, Stuttgart, Germany. Human peritoneal tissue, blood and peritoneal dialysate was collected at time of surgery at Robert-Bosch Hospital. Written informed consent was obtained from Patients according to an approved protocol (#322/2009BO1, Ethik-Kommission, Eberhard-Karls-Universität Tϋbingen, Germany). The approved protocol included a patient information sheet explaining the purpose of the study and the envisioned use of the tissue specimen. All patients agreeing to participate in the study signed, in the presence of an authorized clinical investigator, a detailed written consent form previously approved by the “Ethik-Kommission” of Eberhard-Karls-Universität Tϋbingen, Germany. Patients were provided one day during which to reconsider their written consent. Surgeries included implantation or reimplantation of peritoneal dialysis (PD) catheters or, for EPS patients, enterolyses or peritonectomies. The diagnosis of EPS was based on clinical and radiological findings and on histological criteria that included fibrosis, fibroblast-like cells, exudation, cellularity and its variability, vessel density and its variability, acute or chronic inflammation, hemorrhage, mesothelial hyperplasia, fibrin deposits, presence of vasculopathy, mesothelial denudation, presence of acellular areas, presence of iron and/or calcium deposits, and osseous metaplasia [23], [25], [40]. Shortly after excision, tissue samples were washed in 0.9% saline solution, placed in RNAlater (Ambion, Woodlands, TX) for 12 hours at room temperature, then stored at −80°C. Clinical and laboratory data were recorded for all patients. Clinical data included peritoneal transporter status, peritoneal dialysis efficiency, and number of peritonitis episodes.

### RNA isolation from fresh-frozen (FF) tissue

RNA was isolated from fresh-frozen tissues using Trizol reagent (Invitrogen, Carlsbad, CA). Frozen tissue samples were homogenized in 1 ml Trizol reagent using a rotor-stator homogenizer (Tissue Tearor, BioSpec Products, Bartlesville, OK) for 1 minute. After chloroform extraction of the homogenate, the aqueous RNA-containing supernatant was twice ethanol-precipitated and resuspended in nuclease-free water. RNA concentration was measured by UV spectrophotometry (NanoDrop ND-1000, NanoDrop Technologies, Wilmington, DE). RNA integrity was assessed using the Agilent 2100 Bioanalyzer (Agilent Technologies, Santa Clara, CA).

### cDNA synthesis

Tissue RNA samples were DNAse-treated (DNA-free kit, Applied Biosystems, Carlsbad, CA, Foster City, CA), and 250 ng total RNA from each specimen was reverse-transcribed using the High Capacity cDNA Reverse Transcription Kit (Applied Biosystems) for 10 min at 25°C, 120 min at 37°C, and 5 min at 85°C in a GeneAmp PCR System 2700 thermal cycler (Applied Biosystems). Resultant cDNA samples were diluted 10-fold.

### Quantitative reverse transcriptase polymerase chain reaction (qRT-PCR) for individual gene expression assays

qRT-PCR was performed in duplicate with TaqMan gene expression kits and TaqMan Fast Universal PCR master mix (2x) using the “fast protocol” performed on the 7500 Fast Real-Time PCR System (Applied Biosystems) in bar-coded MicroAmp 96-well plates (Applied Biosystems), per manufacturer's instructions. The gene products analyzed included Thrombospondin 1 (THBS1), Matrix Metalloproteinase 2 (MMP2), Leptin (LEP), Retinol-Binding Protein 4 (RBP4), Runt-Related Transcription Factor 2 (RUNX2), Intercellular Adhesion Molecule 1 (ICAM-1), α Smooth Muscle Actin (ACTA2), Fibronectin 1 (FN1), Collagen 1α1 (Col1a1), and Sulfatase 1 (SULF1). β-actin (ACTB) was used as an endogenous control.

### DNA microarrays

Total RNA was reverse transcribed into cDNA using the Ovation Pico WTA System (NuGen Technologies, San Carlos, CA). cDNA samples were cleaved and 3′-biotinylated using the Encore Biotin Module (NuGen Technologies, San Carlos, CA), yielding biotinylated single-stranded cDNA probes of 50–100 nt in length. The probe mix was hybridized with the GeneChip Human Genome HT U133 Plus PM Array plate (Affymetrix, Santa Clara, CA), presenting >54,000 target probe sets representing 47,000 transcripts encoding >33,000 well characterized human genes. Microarray analysis was conducted by the Beth Israel Deaconess Medical Center Genomics and Proteomics Center according to standard Affymetrix protocol, using a high throughput hybridization and scanning system. The quality of hybridized chips was assessed using Affymetrix guidelines based on PM mean, 3′ to 5′ ratios for beta-actin and GAPDH, and values for spike-in control transcripts. We also monitored sample reproducibility by chip- to-chip correlation and signal-to-noise ratio (SNR) methods for replicate arrays using the bioconductor package, arrayQualityMetrics [41]. All high quality arrays were included for unsupervised and supervised bioinformatics analysis.

To obtain signal values, chips were further analyzed using the Robust Multichip Average (RMA) method in R using Bioconductor and associated packages. RMA performed background adjustment, quantile normalization and final summarization of 11 oligonucleotides per transcript using the median polish algorithm [42]. Unsupervised analysis was performed using Principal Component Analysis (PCA), which projects multivariate data objects onto a lower dimensional space while retaining as much of the original variance as possible [43], [44]. Before PCA, transcripts were filtered to include only those with absolute expression ≥10 in at least 10% of samples. When comparing two groups of samples to identify genes enriched in a given phenotype, i.e. enriched in Uremic vs. both EPS and PD, differentially expressed genes were defined as those with LCB>2, serving as a stringent estimate of the FC [45]. (LCB is defined as the 90% Lower Confidence Bound of the fold change (FC) between the two groups, providing 90% confidence that the actual FC exceeds the threshold LCB).

### Self Organizing Maps (SOM)

Identification of functionally related genes differentially expressed in the profiles of EPS, or in the profiles of both EPS and PD as compared to Uremic, was by self-organizing map (SOM) analysis of the differentially expressed genes. We performed SOM clustering on transcript expression values using Pearson correlation coefficient-based distance metrics and a target of 40 groups. SOM allowed grouping of gene expression patterns into an imposed structure in which adjacent clusters are related, thereby identifying sets of genes that follow common expression patterns (transcription signatures) across different conditions [45], [46].

### Pathways and interactive network analysis

Ingenuity Pathway Analysis (IPA 7.0, Ingenuity Systems, Inc., Redwood City, CA) was used to identify pathways and interaction networks significantly affected by genes with expression changes specifically associated with EPS or with both EPS and PD. The knowledge base of this software consists of functions, pathways and network models derived by systematic exploration of the peer-reviewed scientific literature. IPA analysis calculates a P-value for each pathway according to the fit of user data to the IPA database, using a one-tailed Fisher exact test (http//www.ingenuity.com). Pathways with multiple test-corrected P-values<0.05 were considered significantly affected.

An IPA score [(−log(P value)]>2 indicates a probability >99% that the affected network was not generated at random, but reflects statistically (and biologically) meaningful relationships among a set of genes with specifically altered expression. The ability to rank networks based on relevance to the queried data sets allows network prioritization according to highest predicted impact on the disease process.

### Immunohistochemistry

Deparaffinized, rehydrated tissue sections were incubated in Peroxidase Blocking Solution (S 2023, DAKO, Hamburg, Germany). Immunostaining was performed with a TechMate 500 Plus (DAKO), using a dextran-coated peroxidase-coupled polymer system (Dako REAL™ EnVision™ Detection Kit, Peroxidase/DAB+, Rabbit/Mouse, K 5007, DAKO). The primary antibody was rabbit monoclonal anti-human collagen 1 α 1 (AB292, Abcam, Cambridge, MA) diluted 1∶100 in DAKO S 2022 diluent. Skin tissue from a human non-diabetic leg served as a positive tissue control. Omission of primary antibody served as negative control. Adjacent tissue sections were stained with Hematoxylin and Eosin (H&E).

Semiquantitative analysis of Collagen 1 α 1 immunostaining was performed within defined areas from 0–100 µm and from 100–200 µm inward from the mesothelial tissue edges towards the adventitia, using ImageJ 1.46 (NIH). Color images recorded by an Olympus BX41 microscope equipped with an Olympus DP71 camera were converted to 32-bit black and white. Immunostaining detection threshold was zeroed at background staining intensity, and ranged from 0–255 arbitrary units. Measurements were recorded at three or more discreet locations within each specimen. Scaled intensity values in the region 0–100 µm from the mesothelial edge were divided by the intensity values in the adjacent region 100–200 µm from the edge. The mean intensity ratios for each group were normalized to that of the Uremic group, to which was assigned a relative intensity ratio value of 1.0.

### Statistical Analysis

Clinical data and laboratory values were compared by ANOVA (Sigmaplot 11.0, Systat Software Inc., San Jose, CA). Differences in results were considered statistically significant for P<0.05.

## Results

### Patient characteristics

Tissue samples were available from 8 patients: 4 with encapsulating peritoneal sclerosis (EPS, 2 males and 2 females, of mean age 60.8 yrs and mean PD duration 74±34 months), 2 chronic peritoneal dialysis patients (PD, 1 male and 1 female, of mean age 68.5 yrs and mean PD duration 23 months), and 2 male Uremic patients of mean age 56.5 yrs, as yet undialyzed (Table 1, Table S1). Uremic patients had an estimated glomerular filtration rate (eGFR)<15 mL/min/1,73 m^2^ body surface area. Residual renal function (urine output, RRF) was 275±246 mL in the EPS group, 440 mL for the PD group, and 2000 mL in the Uremic group (p<0.01 Uremic vs. both groups). Additional clinical characteristics are summarized in Table 1 and (for individual patients) Tables S2a and S2b. Serum chemistries are presented in Table S1.

**Table 1:** Patient characteristics.

Clinical data	EPS (4)	PD (2)	Uremic (2)
Age [a]	60.75±12.42	68.50	56.50
Sex	2 m, 2 f	1 m, 1 f	2 m
Time on PD [months]	74.00±33.52	23	-
Kt/V	1.96±0.32	2.41	-
Bacterial peritonitis [# of episodes]	2.25±2.63	0.50	-
24 h urine output [mL]	275.00±246.64	440.00	2000.00
Nicotine abuse	2/4	0/2	2/2
Arterial hypertension	4/4	1/2	2/2
Diabetes of any kind	1/4	2/2	2/2
PD fluids	2 neutral/2 acidic	both neutral	-
Icodextrin use	4/4	2/2	-
**Laboratory values**			
Hemoglobin [g/L]	111.75±7.63	100.00	131.50
Leucocytes [×10^9^/L]	8.79±3.41	4.70	6.40
CRP [mg/dL]	2.97±3.35	1.35	0.30
Creatinine [mg/dL]	3.55±1.84	4.00	5.80
BUN [mg/dL]	86.58±36.40	61.00	173.00
Calcium [mM/L]	2.35±0.10	2.09	2.51
Phosphate [mM/L]	1.45±0.34	1.51	1.45

Clinical data and laboratory values of (n) patients, collected before the surgeries that allowed collection of peritoneal biopsy samples. Values are shown as means ± s.e.m. for the EPS group and as means for the smaller PD and Uremic groups. Smoking history was not stratified by duration. Uremic group 24 h urine output was higher than that of the combined PD and EPS groups (p<0.001). Arterial hypertension was defined as resting arterial blood pressure ≥140/90 mmHg. Acidic PD solutions were lactate-buffered with pH 5.0–5.5. Neutral (multicomponent) solutions were of pH 6.5. Icodextrin status was listed as positive if used at any time during the course of PD.

EPS, Encapsulating peritoneal sclerosis; PD, Peritoneal dialysis; CrP, C reactive protein; BUN, blood urea nitrogen.

### DNA microarray analysis - unsupervised analysis

**Figure 1:**
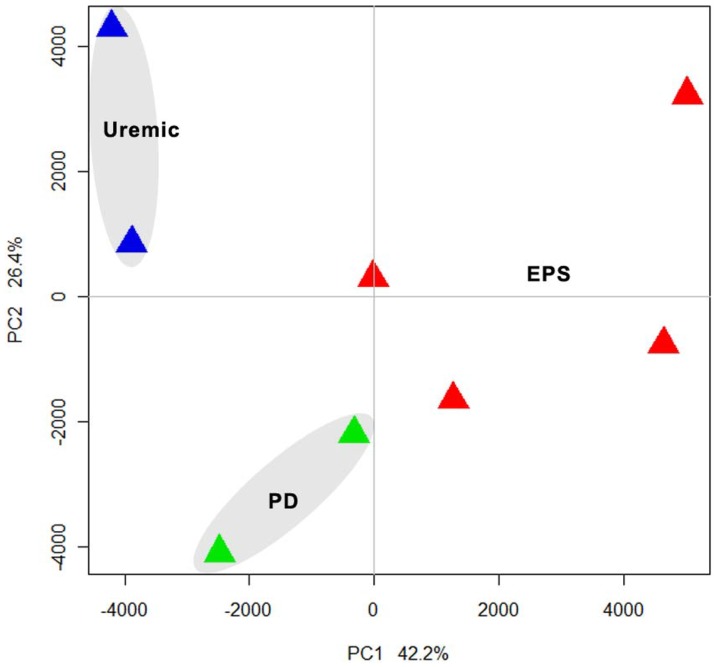
Principal Component Analysis (PCA) of normalized expression data obtained from Uremic, PD and EPS samples. The first component with highest variance (42.2%) is on the X-axis separating Uremic and PD from EPS samples. The second highest (26.4%) is on the Y-axis depicting maximum variation between PD and Uremic samples. The Uremic, PD and EPS samples formed three separate clusters on the PCA plot.

Unsupervised analysis was performed on ∼11,500 transcripts after preprocessing and normalization of high quality DNA expression data. By principal component analysis, we demonstrated that samples separated according to EPS status along primary component (PC) 1, which accounted for 42.2% of the variation between samples. The analysis revealed three distinct clusters of gene expression by principal component analysis (PCA) (Figure 1), consistent with the three clinical groups.

### Identification of genes significantly dysregulated only with EPS or both EPS and PD

**Figure 2:**
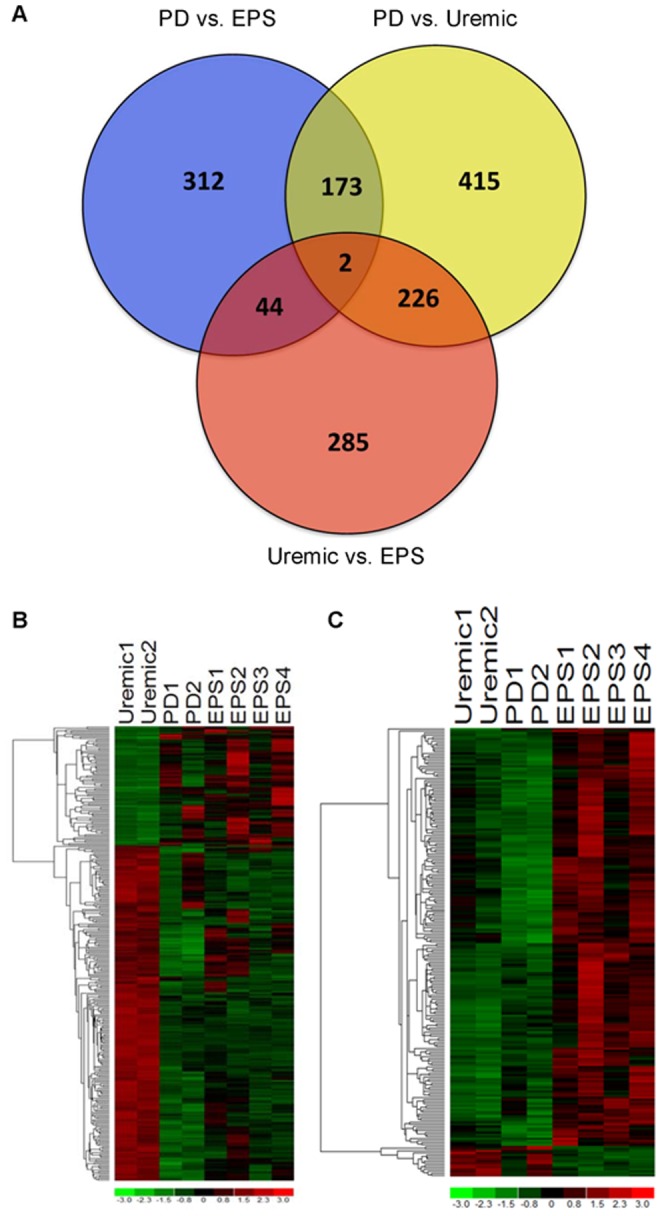
Differentially expressed genes identified from supervised analyses. **A.** Venn diagram comparing significantly differentially expressed genes identified from the pairwise comparisons Uremic vs. PD (yellow), PD vs. EPS (blue), and Uremic vs. EPS (red). The genes were selected using supervised analysis on the basis of the 90% lower confidence bound (LCB) of the fold change (FC) by pairwise comparison of the groups. The analysis was performed on preprocessed data by filtering out low-expressing probes on the basis of absolute intensity (Intensity <10 in 90% of samples). **B.** Heatmap of genes differentially expressed in both EPS and PD groups as compared to Uremic group (P<0.05). **C.** Heatmap of genes differentially expressed only in EPS as compared to both PD and Uremic groups (P<0.05). Columns represent the samples, with rows representing genes. Gene expression levels are presented in pseudocolor (scale −3 to 3), with red and green respectively denoting high and low expression levels.

Supervised analysis applying a 90% lower confidence bound of the fold change (LCB of FC), and expressed as corrected fold-change >2.0, revealed 531 genes differentially expressed between EPS and PD groups, 557 genes differentially expressed between EPS and Uremic groups, and 816 genes differentially expressed between PD and Uremic groups (Figure 2A). Tables S2, S3, S4 present the 50 genes most highly differentially expressed in pairwise comparisons of the three clinical groups. The group of genes upregulated in EPS compared to PD is dominated by genes involved in immunological processes (e.g. MHC class II and associated proteins). The comparison of EPS to the Uremic group featured upregulation of genes encoding collagens and other matrix proteins, and downregulation of lipid metabolism genes such as glycerol-3-phosphate acyltransferase (∼20-fold). In contrast, comparison of PD with Uremic reveals significant upregulation of lipid metabolism genes (20-fold increased glycerol-3-phosphate acyltransferase) and downregulation of matrix metabolism genes, including integrins and collagens.

The Venn diagram of Figure 2A shows that 228 transcripts are differentially expressed in both the comparisons between EPS and the Uremic control group and between PD and Uremic groups, and these represent a candidate transcription signature for peritoneal fibrosis. The Venn diagram also depicts a set of 641 transcripts that are differentially expressed uniquely in EPS as compared to PD or Uremic, and so might include candidate genes linked to the pathological progression from uremic changes, through the simple peritoneal fibrosis of PD, and on to EPS.

When using gene clustering and self-organizing maps (SOM) to detect groups of differentially expressed genes with similar expression patterns, we arbitrarily drew 40 separate maps according to Pearson correlation coefficient-based distance metrics (Figure S1). Evaluation of the maps revealed groups of similarly structured expression patterns, allowing selective merger of patterns depicting specific dysregulation in EPS vs. both PD and Uremic, or dysregulation in both EPS and PD as compared to Uremic. The analysis identified a set of 316 transcripts differentially expressed in both EPS and PD as compared to Uremic (fibrosis signature) (Figure 2B). This analysis also identified another set of 219 transcripts specifically differentially expressed in EPS vs. PD as well as in EPS vs. Uremic (EPS Signature) (Figure 2C).

### Canonical pathways analysis of EPS and Disease Signatures

**Figure 3:**
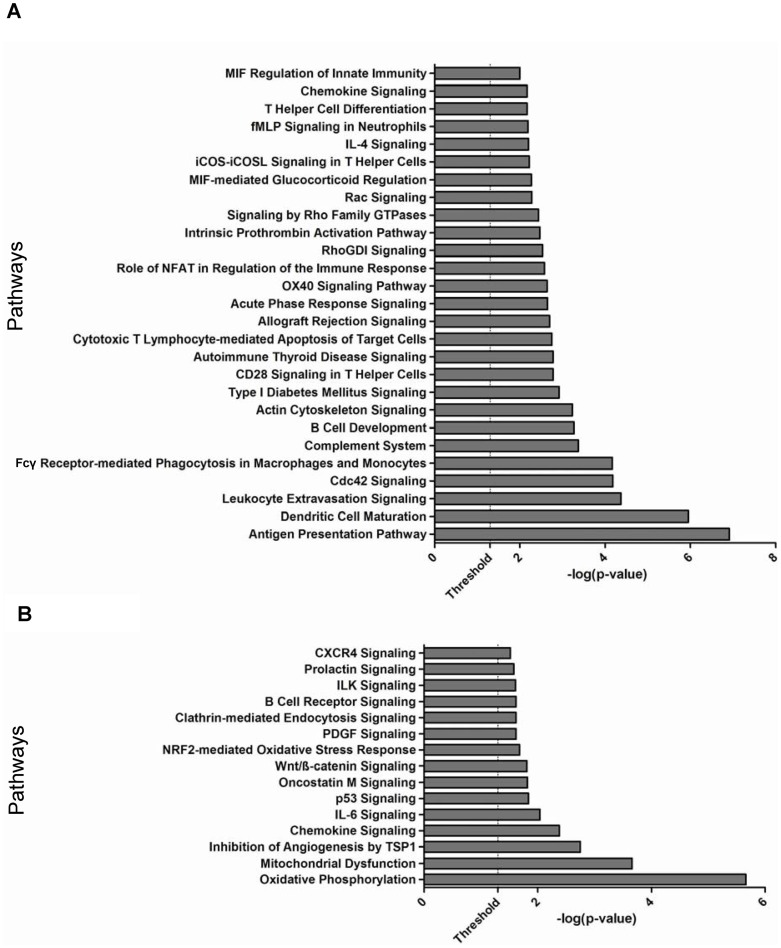
Pathway enrichment analysis of differentially expressed genes. **A.** Genes differentially expressed only in EPS as compared to PD and Uremic groups. **B.** Genes differentially expressed in both EPS and PD groups as compared to the Uremic group. Each bar represents a significantly enriched pathway as determined by Fisher's Exact Test P value [depicted on the X-axis as −log10(P value)]. The analysis for canonical pathways was performed using Ingenuity Systems software (www.ingenuity.com).

We performed canonical pathways enrichment analysis (IPA 7.0) to gain further insight into the functional pathways associated with genes dysregulated only in EPS (EPS signature), and with those dysregulated in both EPS and PD (Disease signature). EPS-associated genes were significantly over-represented (P value<0.05) in pathways related to immune response, inflammation and cytoskeleton signaling, including *“antigen presentation”, “Dendritic cell maturation”, “B cell development”, “complement system”, “chemokine signaling” and “humoral and cellular immunity”* (Figure 3A). Most of the genes in these pathways were upregulated in EPS, suggesting enhanced activity of innate immune and inflammation pathways.

Pathway analysis of the genes differentially expressed in both the EPS vs. Uremic and the PD vs. Uremic comparisons showed significant association with pathways involved in cell signaling, immune response and metabolism, including *“Oxidative Phosphorylation”*, *”Mitochondrial Dysfunction”*, *“Oncostatin M-”*, *“ILK-”*, *“CXCR4-”* and *“PDGF -”* signaling (Figure 3B).

### EPS impacts critical molecules and networks linked to inflammation, immunological response, and cell proliferation

**Figure 4:**
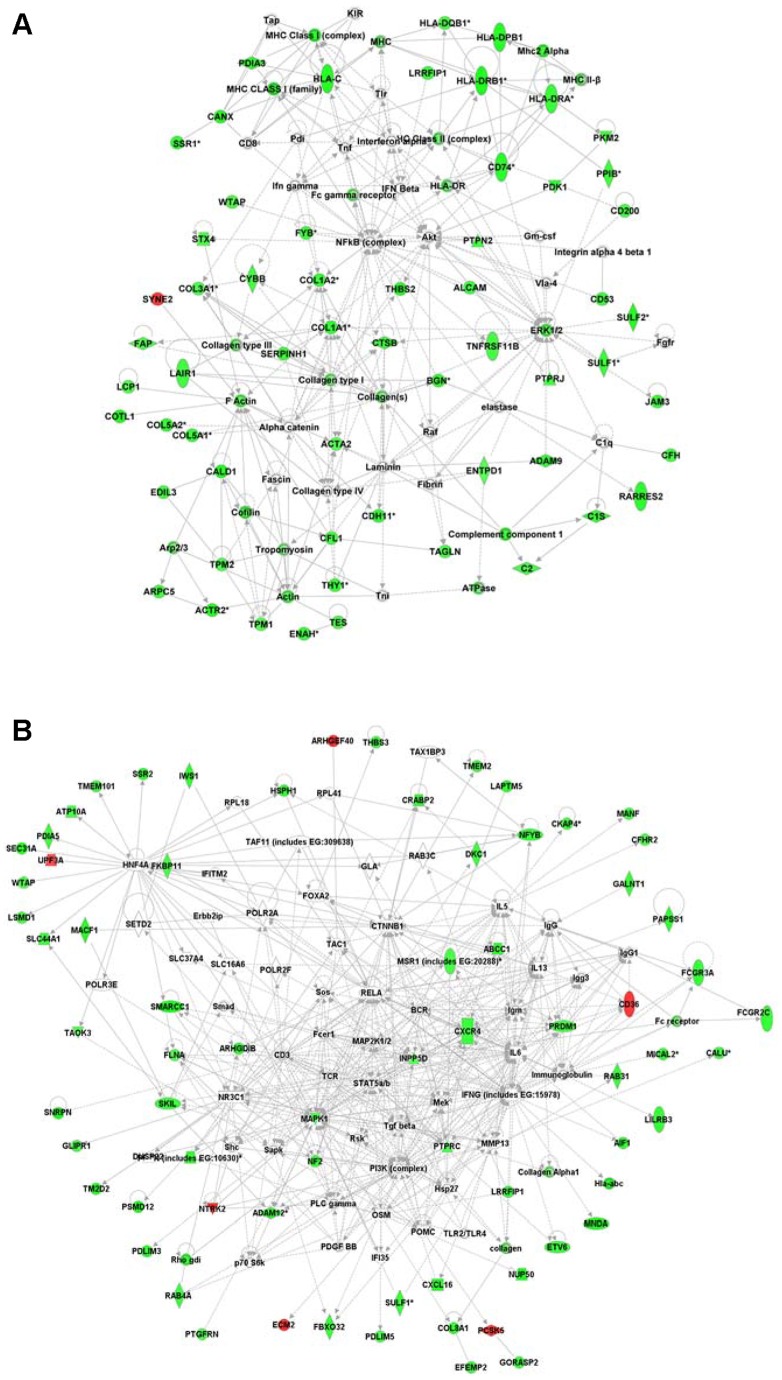
Network representation of cellular functions differentially expressed specifically in EPS but not in PD or Uremic groups. **A.** Network related to cell assembly and organization as well as to connective tissue and skeletal muscle tissue disorders. This network has NF-κB, collagen, and ERK1/2 genes as primary regulatory focus nodes. **B.** Network enriched with genes involved in cellular growth and proliferation, carbohydrate metabolism, and gastrointestinal and immunological diseases. This network has PI3K, IFNγ and MAPK1 genes as primary regulatory focus nodes. Ingenuity Pathways Analysis software was used to generate comprehensive gene networks that merged affected networks of related function. Downregulated genes are shown in green, upregulated genes in red. All networks shown were significantly affected in EPS with a score >15 (−log 10[Fisher's Exact Test]).

To integrate a functional view of critical regulatory molecules associated with EPS, we performed interactive network analysis on the 228 genes specifically dysregulated in EPS tissue specimens (Figure 2C). The *Ingenuity Pathways Analysis* tool was applied to generate interaction networks based on known functional interactions such as protein-protein interactions or gene regulation interactions (Figure 4). The analysis identified 8 different networks that were significantly affected in EPS with a score >20 (−log 10 [Fisher's Exact Test]). Networks of related function were merged to generate more comprehensive networks. Three different networks related to cell assembly and organization and connective tissue and skeletal muscle tissue disorders were merged to generate an integrated view of cell cycle-related dysregulation in EPS tissue (Figure 4A), featuring the NF-κB, collagen, and ERK1/2 genes as major critical regulatory nodes. Another EPS-associated comprehensive network merging subnetworks linked to cell growth and proliferation and to carbohydrate metabolism (as in gastrointestinal and immunological diseases) features the PI3K, IFNγ and MAPK1 genes as major regulatory focus nodes. These regulatory focus nodes are likely critical to network function, such that therapeutic (or pathologic) alteration of expression of these genes predicts perturbation of the entire network.

### EPS and PD both impact critical molecules and networks associated with inflammatory disease, metabolism, cell motility, and cell signaling

**Figure 5:**
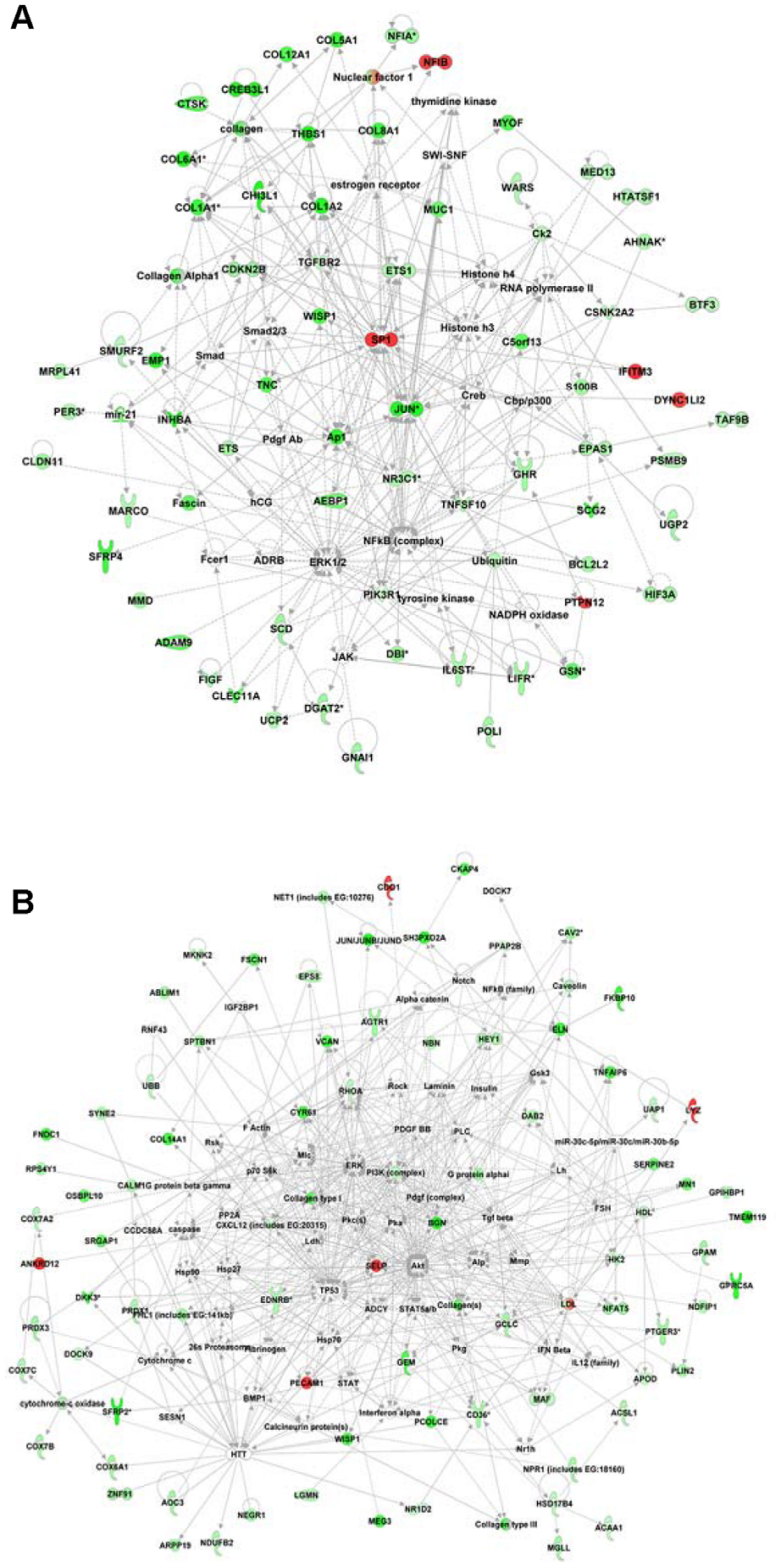
Network representation of the cellular functions affected specifically in both EPS and PD groups, but not in the Uremic group. **A.** Network related to inflammatory and immunological diseases, including NF-κB, JUN, and SP1 genes as primary regulatory focus nodes. **B.** Network enriched with genes involved in lipid metabolism, cellular assembly and movement, and cell death, including PI3K, AKT and TP53 genes as primary regulatory focus nodes. The Ingenuity Pathways Analysis tool was used to generate comprehensive gene networks that merged with affected networks of related function. Downregulated genes are shown in green, upregulated genes in red. Geometric shapes are associated with individual gene products according to Ingenuity definitions. All networks shown were significantly affected in EPS with a score >20 (−log 10[Fisher's Exact Test]).

To gain further insight into functional consequences of genes that are altered both in both EPS and PD as compared to the Uremic group, we again performed IPA interactive network analysis, identifying 8 different networks significantly affected in both EPS and PD compared to the Uremic control group with a significance score >20. Three networks related to inflammatory and immunological diseases were merged to identify critical regulatory genes (Figure 5A). The resulting merged network highlighted NFKB, JUN, SP1 as critical regulatory molecules. The independent merged network in Figure 5B is enriched in genes involved in lipid metabolism, cellular assembly and movement, and cell death, and reveals the PI3K, AKT and TP53 genes as major regulatory focus nodes.

### qRT-PCR Validation of DNA array data

**Figure 6:**
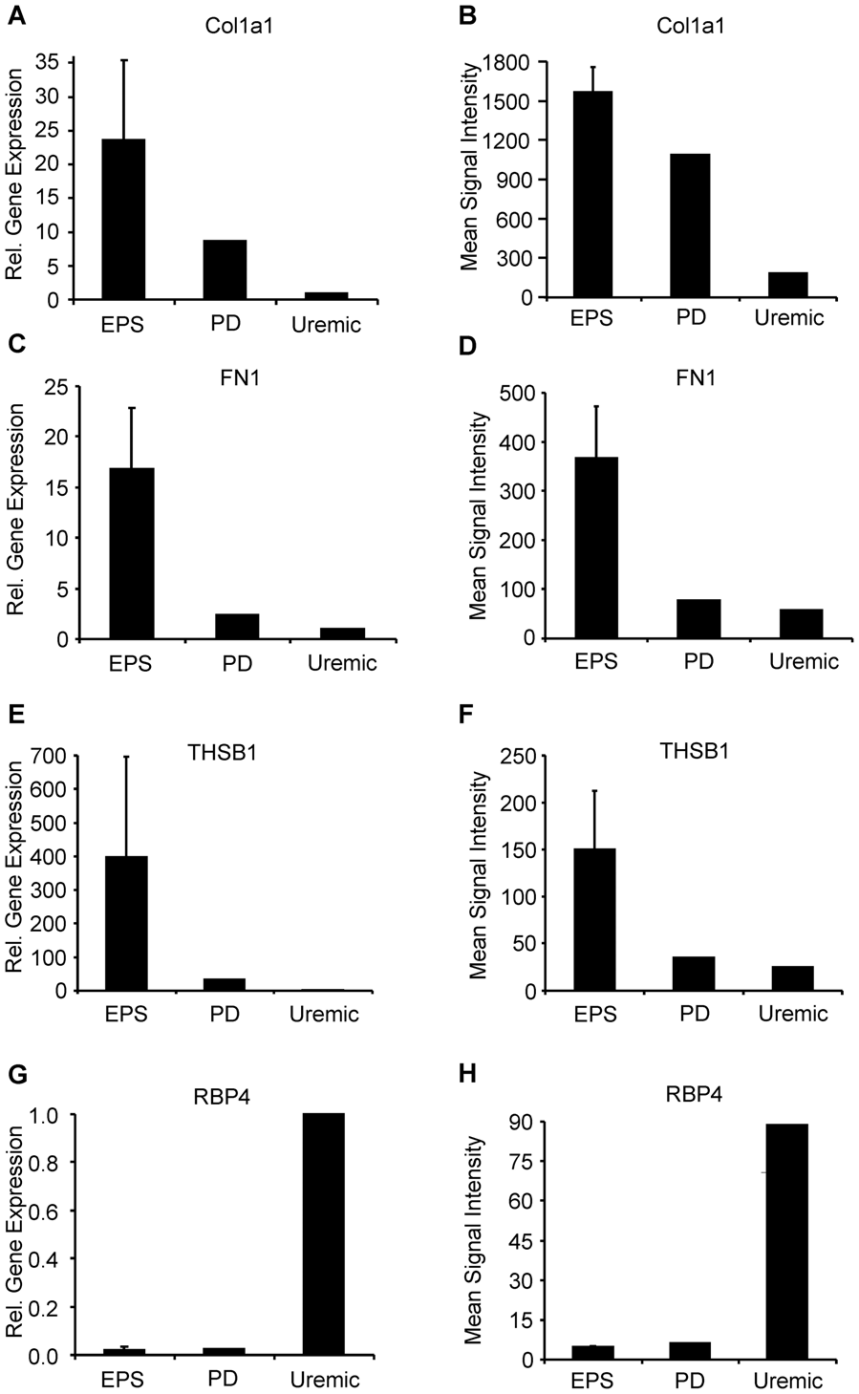
mRNA expression levels of selected gene products determined through qRT-PCR correlate well with corresponding data from the DNA chip array. Collagen 1 α 1 (Col1a1), Fibronectin 1 (FN1), and thrombospondin 1 (THBS1) were highly upregulated in EPS compared to PD and Uremic groups, while retinol-binding protein 4 (RBP4) was highly downregulated in EPS and PD groups compared to the Uremic group. **A.**, **C.**, **E.**, and **G.** Normalized mRNA expression levels determined through qRT-PCR of indicated gene products. Individual gene expression levels were calculated using the equation 2^−ΔCT^ with β-actin (ACTB) as endogenous control. Mean gene expression levels of biological groups were normalized to the Uremic group, defining the relative gene expression in this group as 1.0. **B.**, **D.**, **F.**, and **H.** Mean raw signal intensity of indicated gene products calculated from DNA array probe signals.

Selected cases of differential gene expression were validated by qRT-PCR assays. β-actin was used as an endogenous “control” transcript. The data were analyzed using the 2^−Δ*CT*^ calculation, and then normalized to the Uremic group, for which relative gene expression = 1.0. The results of the DNA chip array are presented as mean raw signal intensities. Col1a1 showed >20-fold upregulation in EPS over Uremic and a 2.5-fold increase in gene expression over PD as judged by qRT-PCR, while the DNA chip array showed >5-fold transcript induction in EPS over Uremic and ∼1.5-fold upregulation over PD (Figure 6). α-Smooth muscle actin (ACTA2), Sulfatase 1 (SULF1) and Intracellular adhesion molecule 1 (ICAM1) exhibited similar expression patterns (Figure S2), with EPS showing the highest levels of transcript induction. Fibronectin 1 (FN1) and Thrombospondin 1 (THBS1) showed yet greater upregulation in EPS compared to EPS and Uremic control groups (Figure 6), whereas Retinol-binding protein 4 (RBP4) (Figure 6) and Leptin (LEP) (Figure S3) were substantially downregulated in both EPS and PD groups compared to the Uremic control group (figure 6). The DNA array results generally correlated well with the qRT-PCR data. However, whereas array data indicated highest expression of Runt-related transcription factor 2 (RUNX2) and Matrix metalloproteinase 2 (MMP2) in the EPS group, qRT-PCR indicated highest expression in the PD group (Figure S4).

### Immunostaining for Collagen 1 α 1 (Col1a1)

**Figure 7:**
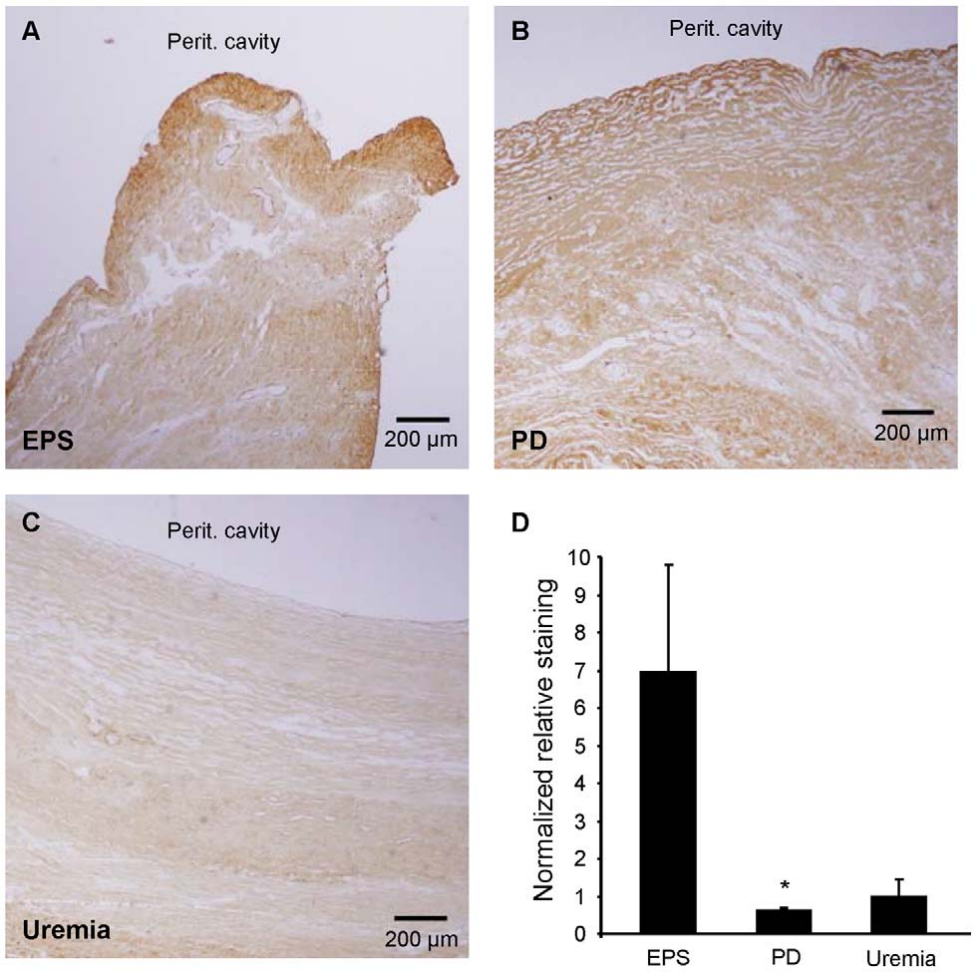
Immunohistochemical expression patterns of collagen 1 α 1 (Col1a1) polypeptide in peritoneal biopsy samples. Peritoneal biopsy sections from (**A.**) EPS, (**B.**) PD, and (**C.**) Uremic groups were analyzed for Col1a1 immunolocalization. Each panel indicates location of peritoneal cavity adjacent to the tissue surface. **D.** Normalized ratios of staining intensities measured in rectangular areas of thickness depth 0–100 µm and 100–200 µm from the peritoneal cavity tissue surface. P<0.05 for EPS vs. PD (One Way ANOVA with Dunn's Multiple Comparison post-test).

To determine protein expression of Col1a1, one of the more highly upregulated genes in EPS, we performed anti-Col1a1 immunohistochemical staining of formalin-fixed, paraffin-embedded tissue sections corresponding to the fresh frozen specimens from which RNA was isolated. EPS and PD tissues showed marked immunostaining for Col1a1, indicating fibrosis. In most of the EPS and PD samples the mesothelium was not detectable. PD sections were strongly and homogeneously stained with Col1a1 throughout the entire specimen. In contrast, EPS samples exhibited pronounced Col1a1 immunostaining predominantly in the area of the (most superficial) submesothelial zone. We quantified the extent of the latter staining pattern by making staining intensity measurements in the submesothelial zone (0–100 µm) and the adjacent, deeper zone (100–200 µm from the surface of the tissue block zone moving towards the adventitial layer). The normalized submesothelial staining intensity of the 0–100 µm zone relative to the deeper 100–200 µm zone was 7-fold higher in the EPS group than in the PD or Uremic groups (Figure 7). Similar zonal analyses with zone thicknesses of 200 and or 500 µm yielded the same result.

## Discussion

EPS is among the most serious complications of chronic PD. Although PD duration seems to be a major risk factor, spontaneous EPS has been reported, and the causes of EPS remain obscure. The factors that govern progression from the common PD-associated condition of simple peritoneal fibrosis to the rare but aggressive condition of EPS are poorly understood. The pilot study presented here is the first comparison of transcriptomes of human peritoneal tissues taken from patients with EPS, PD patients without EPS, and predialytic uremic patients. The three groups show distinct gene expression patterns that allow their separation by principal component and heat map analyses. The results constitute proof-of-principle for a larger-scale study that could generate hypotheses for future EPS research, leading to more rigorous biochemical or cell biologic diagnostic criteria for EPS, and to definition of prognostic markers. Correlation of these and future transcriptome data with proteomics studies from peritoneal tissue and fluid would add further insight into EPS disease pathways and could improve early diagnosis and prevention.

### Patients and tissue samples

The samples examined in this study were the first fresh-frozen tissue samples collected by the Peritoneal Biobank at the Robert-Bosch Hospital in Stuttgart, Germany, as part of a comprehensive database of tissues, serum, whole-blood and peritoneal effluent samples from PD patients, PD patients with EPS, and control patients without and with uremia. Collection of formalin-fixed, paraffin-embedded peritoneal tissue blocks had been initiated previously. The early stage of fresh tissue collection is reflected in the small sample sizes of the current study and the imperfect clinical matching of specimens in terms of PD duration, number of peritonitis episodes, Kt/V, systemic baseline inflammatory state, and use of acidic dialysate likely containing glucose oxidation products [47], . Consistent with their predialytic status, patients of the Uremic control group exhibited higher values of residual urine excretion, blood hemoglobin, serum creatinine, BUN, and calcium than did PD patients (p<0.05 for BUN and calcium) or EPS patients (Table 1).

The limited number and mass of patient samples available for this pilot study also prevented uniformity of dominant tissue histological features of the RNA source tissues within and among clinical groups. Later studies might employ laser capture microdissection to facilitate comparison of histologically similar regions within and among groups.

### Transcriptome patterns and pathways

Transcriptome results exhibited good inter-individual agreement. Unsupervised analysis identified three distinct gene expression patterns consistent with the samples' clinical groupings (Figure 1). Several hundred genes were identified by a moderately stringent criterion as differentially expressed in each pairwise comparison (Figure 2A). Subsets of these genes predictably differentiated the Uremic group from both EPS and PD groups (Figure 2B). However, another subset served to differentiate EPS from both PD and the Uremic group (Figure 2C).

The differentially expressed genes could be organized into canonical pathways that distinguished EPS as a separate group from both the PD and the Uremic groups (Figure 3A), and into pathways that distinguished the Uremic control group from both EPS and PD groups (Figure 3B). The pathways uniting genes upregulated uniquely in EPS are dominated by pathways involved in cellular and humoral defense, including those controlling antigen presentation, dendritic cell maturation, phagocytic function, and leukocyte degranulation. The EPS-upregulated degranulation product, lysozyme, is a component of renal amyloid, but histological evidence of amyloid has not been observed in EPS. Additional serological, immunocyte-related, and innate immune response pathways are also regulated preferentially in EPS (Figure 3A). These results are consistent with the reported contribution of peritoneal complement activation to peritoneal fibrin deposition and early peritoneal fibrosis in a murine model [50], [51]. They are likewise consistent with the reported local proliferation of alternatively activated Mφ2 macrophages without macrophage recruitment from distant sites [52]. These macrophages can secrete proinflammatory mediators during episodes of infectious peritonitis [53], associated with increased risk of EPS. In addition, macrophages express the EPS-upregulated scavenger receptor A (SRA/MSR1), previously associated with fibrotic processes [54]. The elevation in EPS tissue of genes encoding neurotropin receptors and their ligands GDNF, BDNF, neurotrophin-3 and MANF (not shown) is also consistent with increased macrophage activation or function. Coincident upregulation of matrix protease inhibitors such as TIMP1 and SERPINH1 and proteases such as ADAM9 and MM2 is also apparent (not shown).

The pathways shared by EPS and PD groups that differentiate them both from the Uremic group (Figure 3B) feature oxidative mitochondrial metabolism and dysfunction, and a variety of cell signaling pathways. PD is known to promote malnutrition and excessive loss of free amino acids, essential fatty acids [55], and albumin [56], leading to muscle wasting [57]. The downregulation of leptin mRNA in peritoneal tissues from both EPS and PD patients (Figure S2) may reflect malnourishment in chronic PD patients, as serum leptin levels fall during long-term fasting [58]. In contrast, elevated serum and dialysate leptin have been reported during chronic PD [59], [60]. Moreover, treatment of hyperacutely uremic rats with high glucose PD solutions produced hyperleptinemia, and acute exposure of 3T3-L1 adipocytes to high-glucose PD solution increased leptin mRNA levels [61], [62]. The striking downregulation of retinol-binding protein 4 (RBP4) mRNA to <10% of Uremic group levels in both PD and EPS groups has been associated with lean body mass [63] and with ovarian cancer [64], but impaired glucose tolerance and obesity were associated with elevated serum RBP4. RBP4 has not been studied in peritoneal dialysis patients.

### Networks of differentially expressed genes

Figures 4 and 5 provide network views of the interactions of differentially expressed gene products. Figure 4A highlights the importance of multiple collagen isoforms, consistent with the exacerbated fibrotic processes underlying EPS. COL1a1 mRNA showed the highest proportional increase in the EPS group (Figure 6 and Table S3). Col1a1 is not only a major component of fibrotic peritoneal tissue (Figure 6), but has also been reported as a marker of peritoneal fibrocytes together with CD45/PTPRC [65], another gene product upregulated in EPS (Table S2). Also upregulated were matrix proteases and protease inhibitors, and thrombospondin 1 (THBS1), activated through a NF-κB signaling pathway which acts as one of the major regulatory nodes in the presented networks [66]. THBS1 is involved in physiological tissue repair and extracellular matrix remodeling as a regulator of TGF-β-mediated fibrosis [67], and as a proinflammatory and profibrotic factor in chronic kidney disease and kidney fibrosis [68]. Also markedly upregulated was sulfatase 1 (SULF1) mRNA (Figure 6), which like SULF2 modulates the sonic hedgehog (Shh), wingless (Wnt), fibroblast growth factor (FGF), and vascular endothelial growth factor (VEGF) signaling pathways. The highly upregulated Fibronectin 1 transcript (Figure 6) encodes a ubiquitously expressed extracellular matrix protein involved in wound healing [69], [70] and is overexpressed in fibrotic tissues [71], [72], induced in fibroblasts and myofibroblasts, and implicated in glomerular and interstitial renal fibrosis [73].

The myofibroblast marker α-smooth muscle actin (ACTA2) mRNA was also upregulated (Figure S2). ACTA2 has been implicated in TGF-β-induced epithelial-to-mesenchymal transition (EMT) in bronchial cells in a cellular model of asthma [74], and in numerous other models of tissue fibrosis. EMT is believed to play an important role in EPS, especially in postulated mesothelial cell transformation into myofibroblasts [75]. One of the important pathways through which EMT is induced is the ERK1/2 pathway [76], another central regulatory node in the EPS-related networks (Figure 4A). The transcription factor TWIST, upregulated 5-fold in PD and 2.5-fold in EPS (not shown), has been widely implicated as a driver of EMT, most recently in a mouse model of peritoneal fibrosis [77]. Lysyl oxidase, best known for its post-translational lysine hydroxylation of collagen, was recently identified as a transcriptional activator of TWIST and TWIST-mediated EMT [78]. Interestingly, expression of the closely related LOXL2 transcript was 4.2-fold higher in the EPS group than in the PD group (Table S2). LOXL2 transcription can be increased by VEGF and by high glucose [79]. LOXL2 can deaminate Lys4 in histone H3 [80], but at least some of its effects as an inhibitor of differentiation are independent of its enzymatic activity [81].

Several transcriptome studies of rodent peritoneal fibrosis models have recently appeared. Yokoi et al [38] suggested that pleiotrophin plays an important role in both inflammation and fibrosis following acute chlorhexidine gluconate-induced peritoneal injury in mice. Pleiotrophin upregulation was not observed in tissue from our EPS patients compared to chronic PD or Uremic groups. However, observations of elevated procollagen, CCl5, Cxcl16, and Adam12 in that mouse model [38] were also noted in our human EPS specimens. Imai et al described a rat model in which intraperitoneal instillation of high-dose glucose-degradation products led to peritoneal fibrotic changes consistent with a role for EMT in development of peritoneal fibrosis [37]. Le et al induced peritoneal fibrosis in rats via foreign body insertion [36]. As in our observations with human tissue, they observed upregulation of α-smooth muscle actin and other myofibroblast markers, as well as overexpression of profibrotic agents such as TGF-β and CTGF. However, interpretation of mechanistic comparisons between animal models of induced peritoneal fibrosis and PD-associated peritoneal fibrosis and EPS in humans remains difficult.

### Col1a1 IHC Immunohistochemistry

Histochemical expression of collagen I was compared to the increased Col1a1 mRNA levels in EPS tissue. The increased Col1a1 mRNA level in EPS tissue was not paralleled by higher total Col1a1 polypeptide levels, as judged by immunostaining intensity across entire fields of view. Although EPS tissue showed greater Col1a1 expression than did Uremic group tissue, the highest levels of Col1a1 immunostaining were observed in PD tissues. In contrast to the homogeneous distribution of Col1a1 immunostaining across entire PD tissue sections, EPS samples exhibited pronounced intensification of Col1a1 immunostaining in the submesothelial compact zone (Figure 7). This pattern of higher density of fibrous tissue and pronounced accumulation of collagen 1 α 1 protein was also noted in the peritoneal submesothelial compact zone in rats after 4 weeks exposure to PD fluid [82].

### Conclusion

Although EPS remains a poorly understood disease, we have demonstrated a distinct transcriptional pattern of peritoneal tissue from EPS patients that differed from those of PD patients and predialytic uremic patients. The distinct transcriptional patterns of EPS and PD tissues presented here are consistent with the hypothesis that EPS constitutes a separate disease entity, possibly requiring a “second hit”, and not merely an exacerbation of the simple peritoneal sclerosis believed present in all PD patients. However, the data also are consistent with EPS representing a malignant acceleration of PD-associated peritoneal fibrosis influenced by patient-specific risk modifier genes and, possibly, by transplant and/or immunosuppression therapy.

Although our small study does not point to a principal pathogenic mechanism in EPS distinct from that of the simple peritoneal fibrosis of chronic PD, the study does serve as proof-of-principle for future array studies with peritoneal tissue from larger groups of patients. Preliminary data (not shown) indicates that the spectrum and abundance of mRNA isolated from formalin-fixed paraffin-embedded tissues from the same patients studied in this paper were very similar to those of mRNA isolated from fresh-frozen tissue (correlation coefficient >0.91). This suggests the reliability of transcriptome analysis applied to archived paraffin blocks of tissues from EPS and PD patients, especially when combined with laser capture microdissection for comparison of histologically similar regions of tissue. Similar transcriptome studies should be conducted with peritoneal dialysate fluid and cell specimens, and with blood specimens, since accrual of samples will be easier than for peritoneal tissue samples. Moreover, definition of transcriptional signatures may prove to have value for diagnostic monitoring and prognosis. Comprehensive analysis of miRNAs will be important to include in these future studies, since they (or their antagomirs) can be easily envisioned as intraperitoneal therapeutic agents.

## Supporting Information

Table S1 **Individual patient characteristics.** The clinical features and laboratory values represent those of each individual patient's last assessment before the surgical procedure that yielded the tissue samples analyzed in this study. Underlying renal diseases are abbreviated as: IgA, IgA nephropathy; GN, chronic glomerulonephritis; PaI-RPGN, pauci-immune rapid progressive glomerulonephritis; NS, nephrosclerosis; DN, diabetic nephropathy; FSGS, focal segmental glomerulosclerosis; MPO, pANCA-positive (myeloperoxidase) vasculitis. Smoking history was defined as positive regardless of duration. Arterial hypertension was defined as resting arterial blood pressure ≥140/90 mmHg. Acidic PD solutions were lactate-buffered with pH 5.0–5.5. Neutral (multicomponent) solutions were of pH 6.5. Icodextrin status was positive if used at any time during course of PD.
(DOC)

Table S2 **Genes differentially expressed in EPS tissue vs. PD tissue.** **A.** The 50 gene products most highly upregulated in EPS tissue as compared to PD tissue. **B.** All gene products downregulated with corrected FC>2.0 in EPS tissue compared to PD tissue.
(DOC)

Table S3 **Genes differentially expressed in EPS tissue vs. Uremic tissue.** **A.** The 50 gene products most highly upregulated in EPS tissue as compared to Uremic tissue. **B.** The 50 most downregulated gene products in EPS tissue as compared to Uremic tissue.
(DOC)

Table S4 **Genes differentially expressed in PD tissue vs. Uremic tissue.** **A.** The 50 gene products most highly upregulated in PD tissue as compared to Uremic tissue. **B.** All gene products downregulated with corrected FC>2.0 in PD tissue compared to Uremic tissue.
(DOC)

Figure S1 **Selected genomic expression patterns depicting progression from uremia to EPS.** Genes differentially expressed in any group comparison (e.g. Uremic vs. PD, Uremic vs. EPS, PD vs. EPS) were used as the seed set for Self-Organizing Map (SOM) analysis of gene expression. These differentially expressed genes were partitioned to 40 separate maps according to Pearson correlation coefficient-based distance metrics. Selected, biologically interesting SOM maps were manually clustered into 2 biologically relevant categories, each representative of at least two similar SOM patterns: EPS-specific [I, EPS vs. (PD+Uremic), left], and fibrosis-specific [II, (EPS+PD) vs. Uremic, right]. The X-axis arrays individual biological samples, and the Y-axis represents changes in gene expression on a scale from −3 to +3.
(TIF)

Figure S2 **Upregulated gene expression in EPS determined by qRT-PCR correlates well with that measured by DNA microarray.** α-smooth muscle actin (ACTA2) (A,B), sulfatase 1 (SULF1) (C,D), and intra-cellular adhesion molecule 1 (ICAM1) (E,F) are highly upregulated in EPS compared to PD and Uremic groups, as judged both by qRT-PCR data (A,C,E) and by DNA microarray data (B,D,F). Uremic group qRT-PCR expression values were normalized to a value of 1.0, and β-actin mRNA served as endogenous control. DNA array signal intensities are raw probe values.
(TIF)

Figure S3 **Down-regulated gene expression in EPS determined by qRT-PCR correlates well with that measured by DNA microarray.** Leptin (LEP) mRNA levels are greatly downregulated in tissues from EPS and PD patients compared to Uremic group tissues, as judged by normalized qRT-PCR data (A) and by DNA microarray data (B). Uremic group qRT-PCR expression values were normalized to a value of 1.0, and β-actin mRNA served as endogenous control. DNA array signal intensities are raw probe values.
(TIF)

Figure S4 **Two examples of lower correlation between gene expression measured by qRT-PCR and by DNA microarray.** Normalized Runt-related transcription factor 2 (Runx2) mRNA levels measured by qRT-PCR did not significantly differ (A), although DNA microarray suggested that expression in EPS tissue exceeded that in Uremic group tissue (B). Normalized MMP2 mRNA levels measured by qRT-PCR suggested higher levels in EPS and PD tissues than in Uremic group tissues (C), whereas MMP2 levels detected by DNA microarray appeared higher in EPS than in PD or Uremic group tissues (D). Uremic group qRT-PCR expression values were normalized to a value of 1.0, and β-actin mRNA served as endogenous control. DNA array signal intensities are raw probe values.
(TIF)
