# Supplementary material for: Transcriptional Patterns in Peritoneal Tissue of Encapsulating Peritoneal Sclerosis, a Complication of Chronic Peritoneal Dialysis
Source: PLoS One. 2013 Feb 13;8(2):e56389. doi: 10.1371/journal.pone.0056389 (PMC3572070; doi:10.1371/journal.pone.0056389)
Supplement: Table S1 — Individual patient characteristics. The clinical features and laboratory values represent those of each individual patient's last assessment before the surgical procedure that yielded the tissue samples analyzed in this study. Underlying renal diseases are abbreviated as: IgA, IgA nephropathy; GN, chronic glomerulonephritis; PaI-RPGN, pauci-immune rapid progressive glomerulonephritis; NS, nephrosclerosis; DN, diabetic nephropathy; FSGS, focal segmental glomerulosclerosis; MPO, pANCA-positive (myeloperoxidase) vasculitis. Smoking history was defined as positive regardless of duration. Arterial hypertension was defined as resting arterial blood pressure ≥140/90 mmHg. Acidic PD solutions were lactate-buffered with pH 5.0–5.5. Neutral (multicomponent) solutions were of pH 6.5. Icodextrin status was positive if used at any time during course of PD. (DOC) [file pone.0056389.s001.doc]

**Supplemental Table 1. Patient Characteristics.**

| **Clinical data** | **EPS** | | | | **PD** | | **Uremic** | |
| --- | --- | --- | --- | --- | --- | --- | --- | --- |
| Sample ID | 242 | 245 | 251 | 265 | 241 | 248 | 249 | 268 |
| Underlying renal disease | IgA | GN | PaI-RPGN | NS | NS | DN | FSGS, MPO | DN |
| Age [a] | 72 | 70 | 46 | 55 | 68 | 69 | 48 | 65 |
| Sex | F | M | F | M | F | M | M | M |
| Time on PD [months] | 115 | 33 | 76 | 72 | 34 | 12 | - | - |
| Kt/V | 1.62 | 1.75 | 2.15 | 2.30 | 2.70 | 2.11 | - | - |
| Bacterial peritonitis [# of episodes] | 5 | 0 | 4 | 0 | 0 | 1 | - | - |
| 24 h urine output [ml] | 400 | 550 | 0 | 150 | 150 | 730 | 2000 | 2000 |
| Nicotine abuse | No | Yes | Yes | No | No | No | Yes | Yes |
| Arterial hypertension | Yes | Yes | Yes | Yes | Yes | No | Yes | Yes |
| Diabetes mellitus of any kind | No | Yes | No | No | Yes | Yes | Yes | Yes |
| PD fluids | Acidic | Neutral | Acidic | Neutral | Neutral | Neutral | - | - |
| Icodextrin use | Yes | Yes | Yes | Yes | Yes | Yes | - | - |
| **Laboratory values** |  |  |  |  |  |  |  |  |
| Hemoglobin [g/l] | 119 | 101 | 114 | 113 | 84 | 38 | 120 | 143 |
| Leucocytes [GIGA/l] | 4.5 | 8.0 | 12.6 | 10.1 | 4.6 | 4.8 | 7.4 | 5.4 |
| CrP [mg/dl] | 0.6 | 3.5 | 0.3 | 7.5 | 0.1 | 2.6 | 0.5 | 0.1 |
| Creatinine [mg/dl] | 3.6 | 2.9 | 1.9 | 6.1 | 4.5 | 3.5 | 5.2 | 6.4 |
| BUN [mg/dl] | 113 | 86 | 35 | 112 | 84 | 38 | 156 | 190 |
| Calcium [mmol/l] | 2.40 | 2.20 | 2.42 | 2.38 | 2.22 | 1.96 | 2.49 | 2.53 |
| Phosphate [mmol/l] | 1.68 | 1.06 | - | 1.61 | 2.01 | 1.01 | 1.41 | 1.48 |

EPS, Encapsulating peritoneal sclerosis; PD, Peritoneal dialysis; IgA, IgA nephropathy; GN, Chronic glomerulonephritis; PaI-RPGN, Pauci-immune rapid progressive glomerulonephritis; NS, Nephrosclerosis; DN, Diabetic nephropathy; FSGS, Focal segmental glomerulonephritis; MPO, pANCA-positive (myeloperoxidase) vasculitis; CrP, C reactive protein; BUN, blood urea nitrogen
