# Supplementary material for: Transcriptional Patterns in Peritoneal Tissue of Encapsulating Peritoneal Sclerosis, a Complication of Chronic Peritoneal Dialysis
Source: PLoS One. 2013 Feb 13;8(2):e56389. doi: 10.1371/journal.pone.0056389 (PMC3572070; doi:10.1371/journal.pone.0056389)
Supplement: Table S2 — Genes differentially expressed in EPS tissue vs. PD tissue. A. The 50 gene products most highly upregulated in EPS tissue as compared to PD tissue. B. All gene products downregulated with corrected FC>2.0 in EPS tissue compared to PD tissue. (DOC) [file pone.0056389.s002.doc]

**Supplemental Table 2. Comparison EPS vs. PD.**

| **A.** |  |  |  |  |
| --- | --- | --- | --- | --- |
|  | **Symbol** | **Accession** | **Gene Description** |  |
| 1 | PTPRC | Hs.170121.1 | Protein tyrosine phosphatase, receptor type, C | **Corrected FC** |
| 2 | LYZ | Hs2.234734.3 | Lysozyme | 7.69 |
| 3 | HLA-DQB1 | Hs.73931.3 | MHC, class II, DQ beta 1 | 7.49 |
| 4 | MSR1 | g3004959 | Macrophage scavenger receptor 1 | 6.74 |
| 5 | FCGR2C | g2149629 | Fc fragment of IgG, low affinity IIc, receptor for (CD32) | 6.56 |
| 6 | CD74 | Hs2.438626.1 | CD74 molecule, MHC, class II invariant chain | 6.36 |
| 7 | CD14 | g4557416 | CD14 molecule | 5.94 |
| 8 | MS4A6A | g11641258 | Membrane-spanning 4-domains, subfamily A, member 6A | 5.5 |
| 9 | HLA-DQA1/2 | Hs.198253.2 | MHC, class II, DQ alpha 1 /// MHC, class II, DQ alpha 2 | 5.18 |
| 10 | SULF1 | Hs.70823.0 | Sulfatase 1 | 4.97 |
| 11 | SSR4 | g5454089 | Signal sequence receptor, delta | 4.88 |
| 12 | HBA1/2 | g13650073 | Hemoglobin, alpha 1 /// Hemoglobin, alpha 2 | 4.77 |
| 13 | LAPTM5 | Hs.79356.0 | Lysosomal multispanning membrane protein 5 | 4.66 |
| 14 | FCGR2A | g11056051 | Fc fragment of IgG, low affinity IIa, receptor (CD32) | 4.63 |
| 15 | FN1 | Hs.321592.0 | Fibronectin 1 | 4.59 |
| 16 | FCGR3A/B | g10835138 | Fc fragment of IgG, receptor IIIa (CD16a)/IIIb(CD16b) | 4.5 |
| 17 | UXT | g4759297 | Ubiquitously-expressed transcript | 4.49 |
| 18 | ARHGDIB | Hs2.83656.2 | Rho GDP dissociation inhibitor (GDI) beta | 4.48 |
| 19 | CTSB | g4503138 | Cathepsin B | 4.45 |
| 20 | KIAA1109 | Hs2.6606.1 | KIAA1109 | 4.41 |
| 21 | HSPA6 | Hs.3268.0 | Heat shock 70kDa protein 6 (HSP70B') | 4.38 |
| 22 | HLA-DRA | g188255 | MHC, class II, DR alpha | 4.34 |
| 23 | CYBB | Hs.88974.0 | Cytochrome b-245, beta polypeptide | 4.31 |
| 24 | OSBPL8 | Hs.321129.0 | Oxysterol binding protein-like 8 | 4.31 |
| 25 | LOXL2 | g4505010 | Lysyl oxidase-like 2 | 4.22 |
| 26 | CD163 | g4758721 | CD163 molecule | 4.19 |
| 27 | C3AR1 | g1511643 | Complement component 3a receptor 1 | 4.15 |
| 28 | EIF4A1 | g13623224 | Eukaryotic translation initiation factor 4A, isoform 1 | 4.14 |
| 29 | PXDN | Hs.118893.0 | Peroxidasin homolog (Drosophila) | 4.13 |
| 30 | HEG1 | Hs.10491.0 | HEG homolog 1 (zebrafish) | 4.12 |
| 31 | SCAMP2 | g5730030 | Secretory carrier membrane protein 2 | 4.03 |
| 32 | NRP2 | Hs.17778.6 | Neuropilin 2 | 4 |
| 33 | CALU | g2809323 | Calumenin | 3.92 |
| 34 | CCR1 | Hs.301921.0 | Chemokine (C-C motif) receptor 1 | 3.84 |
| 35 | PRKCI | g432273 | Protein kinase C, iota | 3.83 |
| 36 | MACF1 | Hs.108258.1 | Microtubule-actin crosslinking factor 1 | 3.81 |
| 37 | HLA-DPA1 | Hs.914.0 | MHC, class II, DP alpha 1 | 3.79 |
| 38 | MS4A4A | Hs2.325960.4 | Membrane-spanning 4-domains, subfamily A, member 4 | 3.78 |
| 39 | AIF1 | Hs.76364.3 | Allograft inflammatory factor 1 | 3.77 |
| 40 | LCP1 | g189501 | Lymphocyte cytosolic protein 1 (L-plastin) | 3.75 |
| 41 | COL6A1 | Hs.25459.0 | Collagen, type VI, alpha 1 | 3.73 |
| 42 | TNFRSF11B | g4507566 | TNF receptor superfamily, member 11b+C43 | 3.7 |
| 43 | HLA-DRB1/4 | g4504412 | MHC, class II, DR beta 1 /// MHC, class II, DR beta 4 | 3.65 |
| 44 | MNDA | g4505226 | Myeloid cell nuclear differentiation antigen | 3.62 |
| 45 | C1QB | g11038661 | Complement component 1, q subcomponent, B chain | 3.53 |
| 46 | KDELR1 | g5803047 | KDEL endopl. retic. protein retention receptor 1 | 3.53 |
| 47 | ARRB2 | g4757779 | Arrestin, beta 2 | 3.47 |
| 48 | APLP2 | Hs.279518.4 | Amyloid beta (A4) precursor-like protein 2 | 3.47 |
| 49 | TMED4 | Hs.301226.1 | Transmembrane emp24 protein transp. dom. containing 4 | 3.44 |
| 50 | ITGB2 | g4557885 | Integrin, beta 2 (compl. comp. 3 receptor 3 and 4 subunit) | 3.41 |
|  |  |  |  | 3.39 |
| **B.** |  |  |  |  |
| 1 | C21orf34 | Hs.102754.0 | Chromosome 21 open reading frame 34 |  |
| 2 | MAP1LC3C | g13625772 | Microtubule-associated protein 1 light chain 3 gamma | -3.23 |
| 3 | GPRASP1 | g7662129 | G protein-coupled receptor associated sorting protein 1 | -2.5 |
| 4 | HOXD4 | g11024701 | Homeobox D4 | -2.25 |
| 5 | ISYNA1 | Hs.324618.3 | Inositol-3-phosphate synthase 1 | -2.16 |
| 6 | ADAM33 | Hs.173716.0 | ADAM metallopeptidase domain 33 | -2.06 |
|  |  |  |  | -2.02 |
