# Supplementary material for: Transcriptional Patterns in Peritoneal Tissue of Encapsulating Peritoneal Sclerosis, a Complication of Chronic Peritoneal Dialysis
Source: PLoS One. 2013 Feb 13;8(2):e56389. doi: 10.1371/journal.pone.0056389 (PMC3572070; doi:10.1371/journal.pone.0056389)
Supplement: Table S3 — Genes differentially expressed in EPS tissue vs. Uremic tissue. A. The 50 gene products most highly upregulated in EPS tissue as compared to Uremic tissue. B. The 50 most downregulated gene products in EPS tissue as compared to Uremic tissue. (DOC) [file pone.0056389.s003.doc]

Supplemental Table 3. Comparison EPS vs. Uremic.

| **A.** |  |  |  |  |
| --- | --- | --- | --- | --- |
|  | **Symbol** | **Accession** | **Gene Description** | **Corrected FC** |
| 1 | COL1A1 | Hs.172928.0 | Collagen, type I, alpha 1 | 10.56 |
| 2 | CYR61 | g6649848 | Cysteine-rich, angiogenic inducer, 61 | 7.17 |
| 3 | COL5A1 | Hs.146428.2 | Collagen, type V, alpha 1 | 6.98 |
| 4 | COL1A2 | g4502946 | Collagen, type I, alpha 2 | 6.28 |
| 5 | MIR21 | Hs.79572.4 | MicroRNA 21 | 6.08 |
| 6 | COMP | g4557482 | Cartilage oligomeric matrix protein | 5.93 |
| 7 | FN1 | Hs.321592.0 | Fibronectin 1 | 5.76 |
| 8 | BGN | g12803216 | Biglycan | 5.58 |
| 9 | COL3A1 | Hs.119571.0 | Collagen, type III, alpha 1 | 5.37 |
| 10 | MXRA5 | g9280404 | Matrix-remodelling associated 5 | 5.12 |
| 11 | CDH11 | g4502716 | Cadherin 11, type 2, OB-cadherin (osteoblast) | 5 |
| 12 | FAP | g1924981 | Fibroblast activation protein, alpha | 4.96 |
| 13 | ITGBL1 | g4758613 | Integrin, beta-like 1 (with EGF-like repeat domains) | 4.88 |
| 14 | THBS1 | Hs.164226.0 | Thrombospondin 1 | 4.59 |
| 15 | COL5A2 | Hs.82985.0 | Collagen, type V, alpha 2 | 4.55 |
| 16 | ANTXR1 | g8922545 | Anthrax toxin receptor 1 | 4.43 |
| 17 | CLEC11A | g13543291 | C-type lectin domain family 11, member A | 4.39 |
| 18 | ACTA2 | g4501882 | Actin, alpha 2, smooth muscle, aorta | 4.31 |
| 19 | LMO7 | Hs.193380.0 | LIM domain 7 | 4.21 |
| 20 | SLC12A8 | g13375853 | SLC family 12, member 8 | 4.17 |
| 21 | VCAN | Hs.306542.0 | Versican | 4.15 |
| 22 | CALU | g2809323 | Calumenin | 4.13 |
| 23 | INHBA | g186414 | Inhibin, beta A | 4.09 |
| 24 | EDIL3 | Hs.10283.1 | EGF-like repeats and discoidin I-like domains 3 | 3.97 |
| 25 | SERPINE1 | g10835158 | Serpin peptidase inh., clade E, member 1 | 3.97 |
| 26 | RUNX2 | Hs.122116.0 | Runt-related transcription factor 2 | 3.96 |
| 27 | PDLIM5 | Hs.306913.0 | PDZ and LIM domain 5 | 3.89 |
| 28 | PDLIM3 | g3138919 | PDZ and LIM domain 3 | 3.87 |
| 29 | COL11A1 | 4876385 | Collagen, type XI, alpha 1 | 3.81 |
| 30 | THY1 | Hs.125359.0 | Thy-1 cell surface antigen | 3.76 |
| 31 | CTHRC1 | Hs.283713.0 | Collagen triple helix repeat containing 1 | 3.67 |
| 32 | COL8A1 | Hs.114599.0 | Collagen, type VIII, alpha 1 | 3.65 |
| 33 | SULF1 | Hs.70823.0 | Sulfatase 1 | 3.55 |
| 34 | CXCR4 | Hs.89414.1 | Chemokine (C-X-C motif) receptor 4 | 3.47 |
| 35 | MEG3 | Hs.53112.0 | Maternally expressed 3 (non-protein coding) | 3.44 |
| 36 | EGR1 | g4503492 | Early growth response 1 | 3.42 |
| 37 | SFRP2 | Hs.31386.0 | Secreted frizzled-related protein 2 | 3.41 |
| 38 | DKK3 | g5454007 | Dickkopf homolog 3 (Xenopus laevis) | 3.39 |
| 39 | BASP1 | g5453749 | Brain abundant, membrane attached signal protein 1 | 3.36 |
| 40 | SFRP4 | Hs.105700.0 | Secreted frizzled-related protein 4 | 3.34 |
| 41 | TMEM49 | Hs2.397084.1 | Transmembrane protein 49 | 3.33 |
| 42 | SULF2 | Hs.43857.1 | Sulfatase 2 | 3.32 |
| 43 | LOC100132116 | Hs2.377755.1 | Hypothetical LOC100132116 | 3.31 |
| 44 | SERPINE2 | Hs.21858.1 | Serpin peptidase inh., clade E, member 2 | 3.3 |
| 45 | FNDC1 | Hs.297939.3 | Fibronectin type III domain containing 1 | 3.23 |
| 46 | CFH /// CFHR1 | Hs.296941.0 | Complement factor H /// complement factor H-related 1 | 3.21 |
| 47 | ALCAM | Hs.10247.0 | Activated leukocyte cell adhesion molecule | 3.09 |
| 48 | MMP2 | g11342665 | Matrix metallopeptidase 2 | 3.05 |
| 49 | ADAM12 | g13259517 | ADAM metallopeptidase domain 12 | 2.98 |
| 50 | PDPN | g5454097 | Podoplanin | 2.98 |
|  |  |  |  |  |
| **B.** |  |  |  |  |
| 1 | GPAM | Hs.42586.0 | Glycerol-3-phosphate acyltransferase, mitochondrial | -19.74 |
| 2 | KIAA1881 | Hs.11006.0 | KIAA1881 | -18.01 |
| 3 | RBP4 | g8400727 | Retinol binding protein 4, plasma | -17.13 |
| 4 | SCD | g7415720 | Stearoyl-CoA desaturase (delta-9-desaturase) | -15.42 |
| 5 | PCOLCE2 | g7019482 | Procollagen C-endopeptidase enhancer 2 | -13.37 |
| 6 | MGST1 | Hs.790.2 | Microsomal glutathione S-transferase 1 | -13.04 |
| 7 | AKR1C2 | g531159 | Aldo-keto reductase family 1, member C2 | -11.51 |
| 8 | CES1 | g688112 | Carboxylesterase 1 | -11.35 |
| 9 | MMD | g6912507 | Monocyte to macrophage differentiation-associated | -10.24 |
| 10 | SORBS1 | g7661699 | Sorbin and SH3 domain containing 1 | -10.13 |
| 11 | THRSP | Hs.91877.0 | Thyroid hormone responsive (SPOT14 homolog, rat) | -10.08 |
| 12 | CES4 | Hs.76688.1 | Carboxylesterase 4-like | -9.93 |
| 13 | KLHL31 | Hs.131064.0 | Kelch-like 31 (Drosophila) | -9.76 |
| 14 | AKR1C1 | g5453542 | Aldo-keto reductase family 1, member C1 | -9.54 |
| 15 | ADIPOQ | g4757759 | Adiponectin, C1Q and collagen domain containing | -8.98 |
| 16 | LPL | g4557726 | Lipoprotein lipase | -8.92 |
| 17 | FABP4 | Hs.117687.0 | Fatty acid binding protein 4, adipocyte+D39 | -8.78 |
| 18 | FHL1 | g3859848 | Four and a half LIM domains 1 | -8.55 |
| 19 | CD36 | Hs.325823.0 | CD36 molecule (thrombospondin receptor) | -8.4 |
| 20 | LEP | g4557714 | Leptin | -8.15 |
| 21 | PCK1 | g4505638 | Phosphoenolpyruvate carboxykinase 1 (soluble) | -8.08 |
| 22 | GHR | g4503992 | Growth hormone receptor | -8 |
| 23 | CFD | g4503308 | Complement factor D (adipsin) | -7.33 |
| 24 | G0S2 | Hs.95910.0 | G0/G1switch 2 | -7.09 |
| 25 | CIDEC | g11545806 | Cell death-inducing DFFA-like effector c | -6.74 |
| 26 | LPL | Hs.180878.0 | Lipoprotein lipase | -6.73 |
| 27 | ALDH2 | g4502032 | Aldehyde dehydrogenase 2 family (mitochondrial) | -6.44 |
| 28 | DGAT2 | g13537296 | Diacylglycerol O-acyltransferase homolog 2 (mouse) | -6.28 |
| 29 | COPG2IT1 | Hs.6421.0 | COPG2 imprinted transcript 1 (non-protein coding) | -6.08 |
| 30 | NQO1 | g4505414 | NAD(P)H dehydrogenase, quinone 1 | -6.05 |
| 31 | SAA1 /// SAA2 | g13540474 | Serum amyloid A1 /// serum amyloid A2 | -5.93 |
| 32 | GCOM1 | Hs.50841.0 | GRINL1A complex locus | -5.79 |
| 33 | ADH1B | g178099 | Alcohol dehydrogenase 1B (class I), beta polypeptide | -5.61 |
| 34 | SLC16A7 | g4759119 | Solute carrier family 16, member 7 | -5.35 |
| 35 | C10orf116 | g5802975 | Chromosome 10 open reading frame 116 | -5.27 |
| 36 | ALDH3A2 | g1082035 | Aldehyde dehydrogenase 3 family, member A2 | -5.16 |
| 37 | FIGF | g4758377 | c-fos induced growth factor (VEGFD) | -5.15 |
| 38 | BTNL9 | Hs.106771.0 | butyrophilin-like 9 | -5.03 |
| 39 | MOSC1 | g12232404 | MOCO sulphurase C-terminal domain containing 1 | -5.03 |
| 40 | TNFRSF21 | g7706171 | tumor necrosis factor receptor superfamily, member 21 | -4.95 |
| 41 | CAV2 | g4557412 | caveolin 2 | -4.92 |
| 42 | ABCA8 | g6005700 | ATP-binding cassette, sub-family A (ABC1), member 8 | -4.87 |
| 43 | PPP2R1B | Hs.168737.0 | Protein phosphatase 2, reg. subunit A, beta isoform | -4.79 |
| 44 | SPTBN1 | Hs.324648.0 | Spectrin, beta, non-erythrocytic 1 | -4.78 |
| 45 | ASPH | g11878115 | Aspartate beta-hydroxylase | -4.77 |
| 46 | AIFM2 | Hs.117062.0 | Apoptosis-inducing factor, mitochondrion-associated, 2 | -4.75 |
| 47 | EBF1 | Hs.32425.0 | Early B-cell factor 1 | -4.67 |
| 48 | CDKN2B | Hs.44565.0 | Cyclin-dependent kinase inhibitor 2B (p15, inhibits CDK4) | -4.64 |
| 49 | PLIN | g4505884 | Perilipin | -4.62 |
| 50 | APCDD1 | Hs.20665.0 | Adenomatosis polyposis coli down-regulated 1 | -4.53 |
|  |  |  |  |  |
