# Supplementary material for: Transcriptional Patterns in Peritoneal Tissue of Encapsulating Peritoneal Sclerosis, a Complication of Chronic Peritoneal Dialysis
Source: PLoS One. 2013 Feb 13;8(2):e56389. doi: 10.1371/journal.pone.0056389 (PMC3572070; doi:10.1371/journal.pone.0056389)
Supplement: Table S4 — Genes differentially expressed in PD tissue vs. Uremic tissue. A. The 50 gene products most highly upregulated in PD tissue as compared to Uremic tissue. B. All gene products downregulated with corrected FC>2.0 in PD tissue compared to Uremic tissue. (DOC) [file pone.0056389.s004.doc]

**Supplemental Table 4. Comparison PD vs. Uremic.**

|  |  |  |  |  |
| --- | --- | --- | --- | --- |
| **A.** |  |  |  |  |
|  | **Symbol** | **Accession** | **Gene Description** | **Corrected FC** |
| 1 | SCD | g11493551 | Stearoyl-CoA desaturase (delta-9-desaturase) | 23.13 |
| 2 | GPAM | Hs.42586.0 | Glycerol-3-phosphate acyltransferase, mitochondrial | 20.96 |
| 3 | MGST1 | Hs.790.1 | Microsomal glutathione S-transferase 1 | 17.56 |
| 4 | SORBS1 | g7661699 | Sorbin and SH3 domain containing 1 | 15.91 |
| 5 | G0S2 | Hs.95910.0 | G0/G1switch 2 | 15.69 |
| 6 | KIAA1881 | Hs.11006.0 | KIAA1881 | 15.66 |
| 7 | CES1 | g688112 | Carboxylesterase 1 | 14.14 |
| 8 | RBP4 | g8400727 | Retinol binding protein 4, plasma | 13.39 |
| 9 | AKR1C2 | g531159 | Aldo-keto reductase family 1, member C2 | 13.11 |
| 10 | ADIPOQ | g4757759 | Adiponectin, C1Q and collagen domain containing | 13 |
| 11 | CD36 | g180112 | CD36 molecule (thrombospondin receptor) | 12.14 |
| 12 | MMD | g6912507 | Monocyte to macrophage differentiation-associated | 11.98 |
| 13 | LEP | g4557714 | Leptin | 11.18 |
| 14 | CES4 | Hs.76688.1 | Carboxylesterase 4-like | 10.88 |
| 15 | LPL | g4557726 | Lipoprotein lipase | 10.56 |
| 16 | RBP7 | Hs.292718.0 | Retinol binding protein 7, cellular | 10.36 |
| 17 | NQO1 | g12654176 | NAD(P)H dehydrogenase, quinone 1 | 10.34 |
| 18 | FABP4 | g4557578 | Fatty acid binding protein 4, adipocyte | 9.84 |
| 19 | CFD | g4503308 | Complement factor D (adipsin) | 9.77 |
| 20 | SAA1 /// SAA2 | g13540474 | Serum amyloid A1 /// serum amyloid A2 | 9.7 |
| 21 | THRSP | Hs.91877.0 | Thyroid hormone responsive (SPOT14 homolog, rat) | 9.57 |
| 22 | APCDD1 | Hs.20665.0 | Adenomatosis polyposis coli down-regulated 1 | 9.37 |
| 23 | AKR1C1 | g5453542 | Aldo-keto reductase family 1, member C1 | 8.69 |
| 24 | EIF1AY | g13528902 | Eukaryotic translation initiation factor 1A, Y-linked | 8.44 |
| 25 | PPP2R1B | Hs.168737.0 | Protein phosphatase 2, reg. subunit A, beta isoform | 8.42 |
| 26 | FHL1 | g3859848 | Four and a half LIM domains 1 | 8.39 |
| 27 | APOL6 | Hs2.363456.1 | Apolipoprotein L, 6 | 7.94 |
| 28 | THRSP | Hs.91877.0 | Thyroid hormone responsive (SPOT14 homolog, rat) | 7.87 |
| 29 | ASPH | g11991236 | Aspartate beta-hydroxylase | 7.59 |
| 30 | DBI | g181960 | Diazepam binding inhibitor | 7.33 |
| 31 | HIF3A | Hs2.371383.1 | Hypoxia inducible factor 3, alpha subunit | 7.11 |
| 32 | TNFRSF21 | g7706171 | Tumor necrosis factor receptor superfamily, member 21 | 6.98 |
| 33 | LIFR | Hs.23767.2 | Leukemia inhibitory factor receptor alpha | 6.72 |
| 34 | ACSL1 | g12669906 | Acyl-CoA synthetase long-chain family member 1 | 6.7 |
| 35 | BTNL9 | Hs.106771.0 | Butyrophilin-like 9 | 6.33 |
| 36 | CAV1 | g4580417 | Caveolin 1, caveolae protein, 22kDa | 6.19 |
| 37 | CRYAB | g3192942 | Crystallin, alpha B | 6.11 |
| 38 | CIDEC | g11545806 | Cell death-inducing DFFA-like effector c | 5.98 |
| 39 | KLHL31 | Hs.131064.0 | Kelch-like 31 (Drosophila) | 5.93 |
| 40 | PCK1 | g4505638 | Phosphoenolpyruvate carboxykinase 1 (soluble) | 5.87 |
| 41 | DGAT2 | g13537296 | Diacylglycerol O-acyltransferase homolog 2 (mouse) | 5.85 |
| 42 | LYVE1 | Hs.17917.0 | Lymphatic vessel endothelial hyaluronan receptor 1 | 5.71 |
| 43 | MINA | Hs.23294.1 | MYC induced nuclear antigen | 5.69 |
| 44 | RGS5 | g5230675 | Regulator of G-protein signaling 5 | 5.66 |
| 45 | GCOM1 | Hs.50841.0 | GRINL1A complex locus | 5.37 |
| 46 | ANKRD40 | Hs.82590.0 | Ankyrin repeat domain 40 | 5.26 |
| 47 | ATP5G3 | g4502300 | ATP synthase, subunit C3 | 5.24 |
| 48 | CDKN2B | Hs.44565.0 | Cyclin-dependent kinase inhibitor 2B | 5.23 |
| 49 | LIPA | g4557720 | Lipase A, lysosomal acid, cholesterol esterase | 5.19 |
| 50 | CTSC | Hs.10029.1 | Cathepsin C | 5.18 |
|  |  |  |  |  |
| **B.** |  |  |  |  |
| 1 | COL1A1 | Hs2.433191.1 | Collagen, type I, alpha 1 | -5.11 |
| 2 | CLEC11A | g13543291 | C-type lectin domain family 11, member A | -4.05 |
| 3 | LOC100128178 | Hs.135159.0 | Similar to hCG2041313 | -4.02 |
| 4 | ITGBL1 | Hs.301296.1 | Integrin, beta-like 1 (with EGF-like repeat domains) | -3.33 |
| 5 | FSCN1 | g13436196 | Fascin homolog 1, actin-bundling protein | -3.23 |
| 6 | COL1A2 | g4502946 | Collagen, type I, alpha 2 | -3.01 |
| 7 | THSD4 | Hs.170345.0 | Thrombospondin, type I, domain containing 4 | -2.83 |
| 8 | TNFAIP6 | g6005905 | Tumor necrosis factor, alpha-induced protein 6 | -2.66 |
| 9 | SERPINE2 | Hs.21858.2 | Serpin peptidase inhibitor member 2 | -2.64 |
| 10 | IQCA1 | Hs.171077.0 | IQ motif containing with AAA domain 1 | -2.54 |
| 11 | IGDCC4 | Hs.20924.0 | Immunoglobulin superfamily, DCC subclass, member 4 | -2.5 |
| 12 | SNED1 | Hs2.7949.1 | Sushi, nidogen and EGF-like domains 1 | -2.5 |
| 13 | BGN | Hs.821.1 | Biglycan | -2.47 |
| 14 | MEGF6 | Hs.124863.0 | Multiple EGF-like-domains 6 | -2.46 |
| 15 | MXRA5 | g9280404 | Matrix-remodelling associated 5 | -2.46 |
| 16 | NR4A1 | g4504440 | Nuclear receptor subfamily 4, group A, member 1 | -2.25 |
| 17 | ZNF432 | g7662313 | Zinc finger protein 432 | -2.19 |
| 18 | CYR61 | g4504612 | Cysteine-rich, angiogenic inducer, 61 | -2.16 |
| 19 | BASP1 | g5453749 | Brain abundant, membrane attached signal protein 1 | -2.14 |
| 20 | C5orf13 | g4758865 | Chromosome 5 open reading frame 13 | -2.08 |
| 21 | ELN | Hs.9295.0 | Elastin | -2.08 |
| 22 | PDLIM3 | Hs.71719.0 | PDZ and LIM domain 3 | -2.08 |
| 23 | RUNX2 | Hs.122116.0 | Runt-related transcription factor 2 | -2.08 |
| 24 | ADAM9 | Hs2.130633.1 | ADAM metallopeptidase domain 9 (meltrin gamma) | -2.06 |
| 25 | C21orf34 | Hs.102754.0 | Chromosome 21 open reading frame 34 | -2.06 |
| 26 | COL27A1 | Hs.284394.1 | Collagen, type XXVII, alpha 1 | -2.01 |
