# Supplementary figures and images for: Transcriptional Patterns in Peritoneal Tissue of Encapsulating Peritoneal Sclerosis, a Complication of Chronic Peritoneal Dialysis
Source: PLoS One. 2013 Feb 13;8(2):e56389. doi: 10.1371/journal.pone.0056389 (PMC3572070; doi:10.1371/journal.pone.0056389)

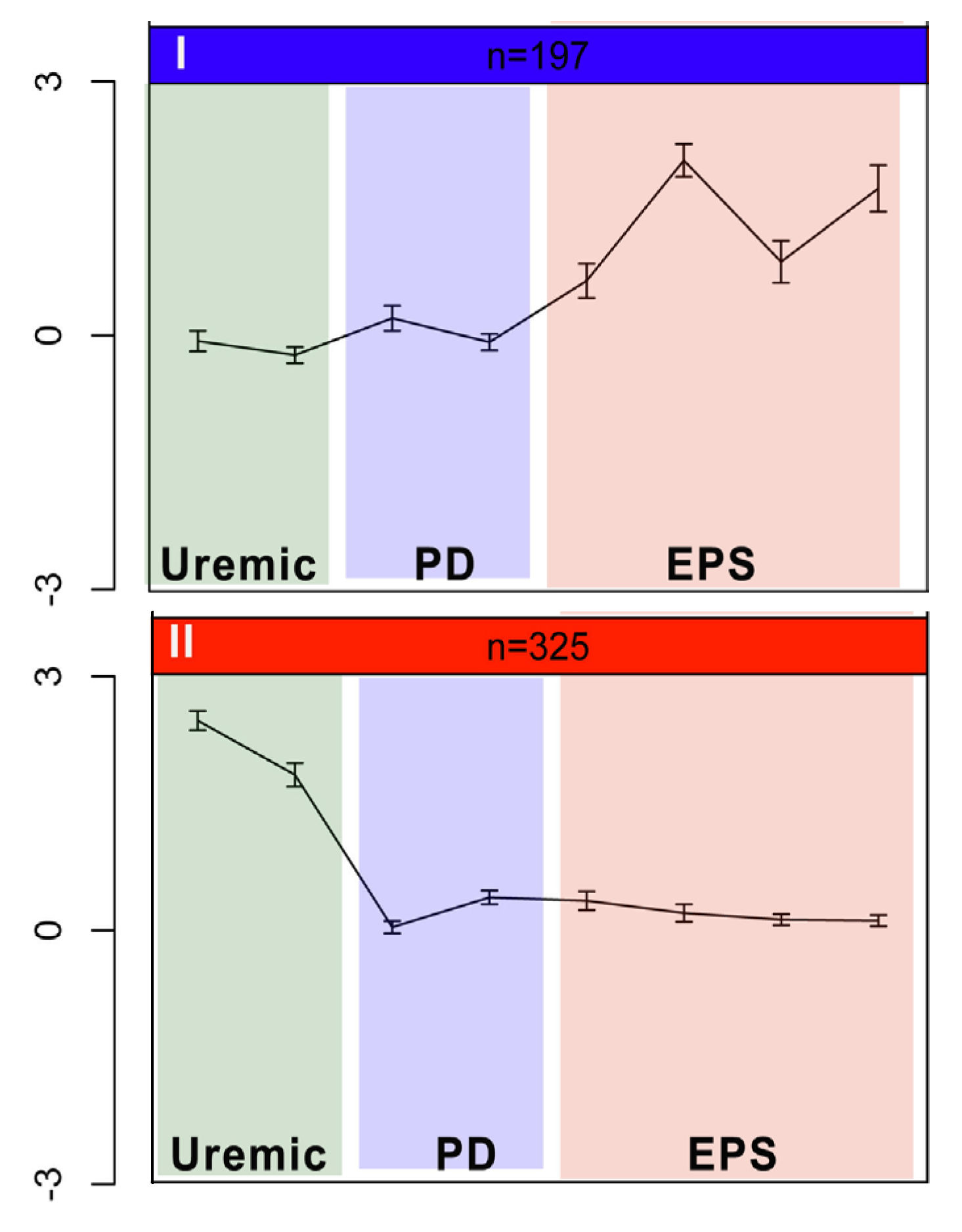

Supplement: Figure S1 — Selected genomic expression patterns depicting progression from uremia to EPS. Genes differentially expressed in any group comparison (e.g. Uremic vs. PD, Uremic vs. EPS, PD vs. EPS) were used as the seed set for Self-Organizing Map (SOM) analysis of gene expression. These differentially expressed genes were partitioned to 40 separate maps according to Pearson correlation coefficient-based distance metrics. Selected, biologically interesting SOM maps were manually clustered into 2 biologically relevant categories, each representative of at least two similar SOM patterns: EPS-specific [I, EPS vs. (PD+Uremic), left], and fibrosis-specific [II, (EPS+PD) vs. Uremic, right]. The X-axis arrays individual biological samples, and the Y-axis represents changes in gene expression on a scale from −3 to +3. (TIF) [file pone.0056389.s005.tif]
